# Fabrication of a Smart Fibrous Biomaterial That Harbors an Active TGF-β1 Peptide: A Promising Approach for Cartilage Regeneration

**DOI:** 10.3390/biomedicines11071890

**Published:** 2023-07-03

**Authors:** Aglaia Mantsou, Eleni Papachristou, Panagiotis Keramidas, Paraskevas Lamprou, Maria Pitou, Rigini M. Papi, Katerina Dimitriou, Amalia Aggeli, Theodora Choli-Papadopoulou

**Affiliations:** 1 Laboratory of Biochemistry, School of Chemistry, Faculty of Sciences, Aristotle University of Thessaloniki, University Campus, 54124 Thessaloniki, Greece; mantsouav@chem.auth.gr (A.M.); epapachristou@chem.auth.gr (E.P.); pankerdim@chem.auth.gr (P.K.); pa.lamprou@yahoo.com (P.L.); margeopit@chem.auth.gr (M.P.); rigini@chem.auth.gr (R.M.P.); 2 Laboratory of Chemical Engineering A’, School of Chemical Engineering, Faculty of Engineering, Aristotle University of Thessaloniki, University Campus, 54124 Thessaloniki, Greece; katdimdim@cheng.auth.gr (K.D.); aggeli@cheng.auth.gr (A.A.)

**Keywords:** cartilage regeneration, smart biomaterials, chondrogenesis, tissue engineering, TGF-β1 peptide, elastin-like polypeptides, silk fibroin, mussel-foot adhesive protein

## Abstract

The regeneration of articular cartilage remains a serious problem in various pathological conditions such as osteoarthritis, due to the tissue’s low self-healing capacity. The latest therapeutic approaches focus on the construction of biomaterials that induce cartilage repair. This research describes the design, synthesis, and investigation of a safe, “smart”, fibrous scaffold containing a genetically incorporated active peptide for chondrogenic induction. While possessing specific sequences and the respective mechanical properties from natural fibrous proteins, the fibers also incorporate a Transforming Growth Factor-β1 (TGF-β1)-derived peptide (YYVGRKPK) that can promote chondrogenesis. The scaffold formed stable porous networks with shear-thinning properties at 37 °C, as shown by SEM imaging and rheological characterization, and were proven to be non-toxic to human dental pulp stem cells (hDPSCs). Its chondrogenic capacity was evidenced by a strong increase in the expression of specific chondrogenesis gene markers *SOX9*, *COL2*, *ACAN*, *TGFBR1A,* and *TGFBR2* in cells cultured on “scaffold-TGFβ1” for 21 days and by increased phosphorylation of intracellular signaling proteins Smad-2 and Erk-1/2. Additionally, intense staining of glycosaminoglycans was observed in these cells. According to our results, “scaffold-TGFβ1” is proposed for clinical studies as a safe, injectable treatment for cartilage degeneration.

## 1. Introduction

Hyaline cartilage is found in joints where it creates smooth surfaces coated with synovial fluid and facilitates the transmission of loads with low friction [1]. Its abundant extracellular matrix consists mostly of large aggrecan/hyaluronic acid complexes that attract large amounts of water osmotically, and collagen type II fibers which provide tensile strength and resistance to shear stress [2]. Although it receives strong mechanical pressures, articular cartilage has low intrinsic capacity for healing and regeneration because of the lack of blood vessels, lymphatic vessels, and nerves [2]. Cartilage defects, as in osteoarthritis, are a common cause of disability worldwide, affecting the majority of the population over 60 years [3]. OA is identified by gradual degeneration of the joint cartilage leading to pain and swelling [4] and, due to the tissue’s low regenerative capacity, clinical treatments for OA focus mainly on the suppression of inflammation and pain relief [1]. To address these issues, studies in the field of biomedical engineering concentrate on the creation of non-toxic, injectable biomaterials that can attract autologous cells and induce tissue regeneration.

Biomaterials that are intended for use as scaffolds for tissue regeneration need to be non-toxic, non-inflammatory, and biodegradable, while also able to provide mechanical support and biological signals to the cells [5]. Silk-elastin-like polymers (SELPs), recombinant polypeptides that are composed of tandem repetitions of a silk fibroin-derived peptide (GAGAGS) and an elastin-derived peptide (VPGXG), have been proven to meet these criteria [6,7,8]. The silk domains, which are inspired from *Bombyx mori* (silkworm) silk fibroin, acquire a formation rich in beta-sheets that provide impressive tensile strength [9], while the domains inspired from human elastin are characterized by reversible phase transition into elastomeric beta-spirals above a certain temperature [10]. The structure and properties of SELPs can be tailored precisely, by controlling the length, number, and sequence of the peptide repeats, by altering the X residue and the silk/elastin ratio, and by introducing crosslinking sites [9]. Silk-elastin hydrogels with RGD motifs from fibronectin have been shown to promote chondrocyte growth and deposition of glycosaminoglycans in vitro and in vivo [8].

Mussel foot adhesive proteins (MFPs) have been studied extensively as a material that enhances scaffold attachment to surfaces. These proteins, which are found in the adhesive plaques of mussels of the genus *Mytilus*, have a high percentage of DOPA (Y*) residues in repetitive sequences such as “AKPSYPPTYK”, which are responsible for stable adhesion to wet surfaces [11,12,13]. Multiple studies have verified that MFPs do not induce toxicity to human cells, on the contrary, MFP-coated scaffolds improve cell attachment and proliferation with high efficacy, and even support cell differentiation [11,14,15,16]. Interestingly, in recent research, highly adhesive hydrogels containing alginate, regenerated silk fibroin, chondroitin sulphate, and dopamine promoted the migration, proliferation, and differentiation of bone marrow stem cells (BMSCs) into chondrocytes and improved cartilage defects in rat knees [13].

In recent research conducted in our laboratory, a scaffold composed of elastin, silk, and mussel peptide repeats with a genetically adapted Bone Morphogenetic Protein-2 peptide (scaffold-BMP2) was synthesized de novo and was shown to promote osteogenesis in human dental pulp stem/stromal cells (hDPSCs) in 21 days by inducing BMP-2 signaling pathways. For enhancement of cell attachment and proliferation, the biomaterial also contained genetically embedded peptides from extracellular matrix glycoproteins. The scaffold was a semi-rigid three-dimensional network with strong shear-thinning properties and was proven to be non-toxic towards the cells [17].

In this project, we took this research a step further, to synthesize a similar scaffold with an embedded peptide sequence from Transforming Growth Factor-β1 (TGF-β1). The peptide “YYVGRKPK” from the carboxyterminal domain of the TGF-β1 polypeptide chain has been shown to enhance chondrogenesis in hDPSCs when added in the culture medium, without leading chondrocytes to a hypertrophic phenotype (Pitou et al., 2023, submitted for publication) [18]. Taking into account these results, we synthesized an elastin-silk-mussel scaffold with cell-attachment peptides and a TGF-β1 peptide (scaffold-TGFβ1). The material was found to possess similar mechanical properties to the scaffold-BMP2 that was produced in our previous work [17], and enhanced cell viability after 14 days of culture. hDPSCs cultured on the scaffold-TGFβ1 had increased expression of chondrogenesis markers, phosphorylation of key signaling proteins (Smad-2 and Erk-1/2) and deposition of glycosaminoglycans, as well as altered cell morphology, after 21 days.

## 2. Materials and Methods

### 2.1. De Novo Synthesis of a Gene Encoding the Polypeptide [TGF-β1 Peptide—(ELP_10_-Silk_2_-Mussel_15_)_2_-Mussel-6xHis]

The DNA building blocks by PCR amplification use sets of semi-complementary primers [10]. The synthesis of the DNA sequences for building blocks “ELP”, “Silk”, and “Mussel” have been described in detail in our previous work [17] (Table S1). The DNA sequence for the building block “TGF-β1 peptide” was synthesized with the same protocol, as described in Mantsou et al. (2023) [17]. The primer sequences for the building block “TGFβ1 peptide” are listed on Table S2 and the composition and conditions of the reaction are listed on Tables S3 and S4. The “TGFβ1 peptide” DNA sequence was cloned into a pET29c expression vector, as it has described previously [17]. The assembly of the final gene sequence was performed with the PRe-RDL method (recursive directional ligation by plasmid reconstruction) [10]. The synthesis of the DNA construct “(ELP_10_-Silk_2_-Mussel_15_)_2_-Mussel-6xHis” has been shown previously [17].

In this work, the plasmids “pET29c-TGF-β1 peptide” and “pET29c-[(ELP_10_-Silk_2_-Mussel_15_)_2_-Mussel-6xHis]” were digested with BglI (New England Biolabs, Ipswich, MA, USA) then the linear plasmid “pET29c-TGF-β1 peptide” was digested with type IIS restriction endonuclease AcuI (New England Biolabs, Ipswich, MA, USA), while the linear pET29c-[(ELP_10_-Silk_2_-Mussel_15_)_2_-Mussel-6xHis] was digested with BseRI (New England Biolabs, Ipswich, MA, USA) [17]. The desired fragments were purified from 1% *w*/*v* agarose gels and ligated with T4 DNA ligase (New England Biolabs, Ipswich, MA, USA), so that the inserts “TGF-β1 peptide” and “(ELP_10_-Silk_2_-Mussel_15_)_2_-Mussel-6xHis” were linked sequentially.

### 2.2. Overexpression and Purification of the Polypeptide [TGF-β1 Peptide—(ELP_10_-Silk_2_-Mussel_15_)_2_-Mussel-6xHis]

For the overproduction of the polypeptide “TGF-β1 peptide-(ELP_10_-Silk_2_-Mussel_15_)_2_-Mussel-6xHis]”, competent *E. coli* BL21 (BE3) bacterial cells were transformed with the plasmid “pET29c-[TGF-β1 peptide-(ELP_10_-Silk_2_-Mussel_15_)_2_-Mussel-6xHis]” and overexpression in large-scale cultures (4 L) was carried out as described previously [17], with the following modification: IPTG was added when the culture reached OD_600_ = 0.4. After 4 h, the cells were collected by centrifugation and lysed by sonication and the polypeptide was purified from the cell extract by nickel affinity chromatography under denaturing conditions with the protocol that has been described in depth before [17]. Samples from all the purification steps were dialyzed against ddH_2_O, to remove guanidine hydrochloride, and were subjected to SDS-PAGE.

### 2.3. Crosslinking and Porous Scaffold Manufacturing

As previously described [17], the isolated polypeptides were crosslinked with the bi-functional, lysine-specific cross-linker “hexamethylene diisocyanate (HDI)”. The utilization of HDI in a mixture of organic solvents DMSO and DMF ensured the inhibition of isothiocyanates hydrolysis that could lead to potential side reactions. Afterwards, the crosslinked polymers were dialyzed against ddH_2_O to remove organic solvents and lyophilized. Salt-leaching with NaHCO_3_ at a weight ratio “polymer:salt” 1:10 was used for the formation of porous scaffolds [17,19].

### 2.4. Rheological Measurements

For the rheological evaluation of scaffolds, a thermostated at 0.1 °C., stress-controlled, AR-G2 rheometer by TA Instruments (New Castle, DE, USA) was utilized. Rheological measurements were performed in each case with a fresh sample to avoid any pretreatment effect unless otherwise stated: dynamic oscillatory strain, frequency, and time sweeps in the linear viscoelastic region (LVR), from which values of elastic (*G*′) and viscous (*G*″) moduli were obtained. Furthermore, steady-state flow steps were performed to calculate dynamic viscosity (η) as a function of shear rate. Calibration samples (standard oils) were determined to be within 5% of their expected levels, verifying the instrument’s reliability.

A total of 6 mg/mL suspensions of crosslinked or uncrosslinked scaffolds in DMEM were prepared. Strain sweeps were performed at 37 °C, with application of 0.01–100% strain at 1 Hz frequency, to determine the LVR. Then, time sweeps were carried out within the LVR (3% strain) on the crosslinked and uncrosslinked biomaterials at 37 °C for 150 s. Temperature sweeps were performed on the crosslinked biomaterial at 10–40 °C and rotational flow measurements at both states of the materials (crosslinked and uncrosslinked) at 37 °C. All measurements were performed with different aliquots of the same sample to avoid pre-shearing effects and repeated with multiple new sample preparations to exhibit reproducibility. Overall, >20 separate scaffold samples were studied in detail rheologically and their consistent, typical behavior is presented here with representative data.

### 2.5. Imaging of Surface Morphology by Scanning Electron Microscopy (SEM)

Salt-leaching and lyophilization were applied to fabricate porous scaffolds on borosilicate glass coverslips (VWR International, Radnor, PA, USA), as previously described [17]. The scaffolds were carbon coated and observed under SEM (JEOL J.S.M. 840A, Tokyo, Japan) at the Electron Microscopy and Structural Characterization of Materials Laboratory of the Department of Physics at Aristotle University of Thessaloniki.

### 2.6. Culture of Human Dental Pulp Stem Cells on the Scaffolds

Human dental pulp stem cells (hDPSCs) were kindly provided by Associate Professor A. Bakopoulou from the School of Dentistry, Aristotle University of Thessaloniki. The cells had been harvested from the third molars of young healthy donors, between the ages 18–24, with the enzymatic dissociation method described in Bakopoulou et al. (2015) [20]. The collection of the samples was executed in accordance with all the relevant guidelines and regulations and had been approved by the Institutional Review Board of the Aristotle University of Thessaloniki (Nr. 66/18-06-2018). All the donors signed an informed consent form.

hDPSCs were maintained in α-MEM supplemented with 15% *v*/*v* fetal bovine serum (FBS), 100 U/mL penicillin, 100 g/mL streptomycin, and 100 mM L-ascorbic acid phosphate (complete α-ΜΕΜ) at 37 °C and 5% CO_2_. Six-well plates were coated with porous scaffolds as described in Section 2.5, sterilized by UV irradiation for 1 min, and then incubated at 37 °C for 24 h. The sterilization method and duration were chosen based on prior work [17] and unpublished animal trials in which it was shown to be efficient and brief enough to minimize detrimental effects on protein structure [19,20]. Cell culture media including a protease inhibitor cocktail for cell culture (Millipore Sigma, Burlington, MA, USA) was applied to the scaffolds for 30 min at 37 °C and 5% CO_2_. Cells were seeded at a density of 2 × 10^5^ cells/well onto the scaffolds and in empty wells as a control. The differentiation studies were carried out in triplicates on 6-well plates. After an overnight incubation, the culture media was changed to chondrogenesis medium, and the relevant wells were treated with 10 ng/mL TGF1 peptide “YYVGRKPK” (GeneCust, Boynes, France). Cells were cultured for 21 days with media replaced every 2–3 days.

All culture media and reagents were purchased from ThermoFisher Scientific (Waltham, MA, USA).

### 2.7. MTT Assay

The 3-(4,5-dimethylthiazol-2-yl)-2,5-diphenyltetrazolium bromide (MTT) assay was used to assess the viability of eukaryotic cells. The scaffolds were plated in 96-well plates at two concentrations, 1 mg/mL and 5 mg/mL, and sterilized according to the instructions in Section 2.6. Afterwards, they were rinsed with 1× PBS and incubated for 30 min at 37 °C and 5% CO_2_ in complete α-MΕΜ with protease inhibitor cocktail (Millipore Sigma, Burlington, MA, USA). Following that, hDPSCs (10^4^ cells/well) were seeded onto the scaffolds and in empty wells and incubated for 3, 7, or 14 days. MTT labelling reagent (Millipore Sigma, Burlington, MA, USA) was added at a final concentration of 0.5 mg/mL in 1× PBS to evaluate cell viability and the plates were incubated at 37 °C and 5% CO_2_ for 3 h. The formazan crystals were dissolved in 100% DMSO at 37 °C for 45 min. The absorbance at 570 nm [blank (DMSO)] and at 630 nm (reference filter) was determined using a microplate reader.

### 2.8. BrdU Assay

The BrdU (5-bromo-2-deoxyuridine) assay (Millipore Sigma, Burlington, MA, USA) was performed to compare the proliferation rate of hDPSCs grown on scaffold-TGFβ1 to that of control cells. The 96-well plates were coated with 1 mg/mL sterilized scaffold-TGFβ1 as described in Section 2.6. After that, it was gently washed with pre-warmed (37 °C) 1× PBS and incubated in fresh complete medium with protease inhibitor cocktail (Millipore Sigma, Burlington, MA, USA) for 30 min at 37 °C and 5% CO_2_. hDPSCs were seeded on the scaffold at a density of 10^4^ cells/well. After 12 h, the medium was replaced in half of the samples by chondrogenesis medium. As a positive control, we used cells treated either with chondrogenesis medium or complete medium α-ΜΕΜ with 10 ng/mL TGFβ1 peptide. After 24 h, 48 h, 72 h, 96 h, and 120 h, 1× BrdU labelling solution was added to each well, incubated for 4 h at 37 °C and 5% CO_2_, and the detection was performed according to the manufacturer’s instructions. Afterwards, the cells were incubated in fixative/denaturing solution for 30 min and then with 1× anti-BrdU antibody solution for 60 min at 25 °C. After the washes, 1× anti-mouse IgG-HRP conjugate was added for 30 min at room temperature. According to the manufacturer’s protocol, 100 μL tetra-methylbenzidine (substrate solution) was added in each well and the plates remained in the dark at room temperature for 9 min. Then, 100 μL of stop solution (2.5 N sulfuric acid) was added and the absorbance was measured in each well using a microplate reader at 450 nm with a reference filter at 690 nm. Internal controls for this test were cell-free and BrdU-free wells and their OD values were used as a blank (negative control) and as a background control (positive control). The experiment was carried out in hexaplicates.

### 2.9. Alcian Blue Staining Assay

Glycosaminoglycans in the extracellular matrix were stained with Alcian Blue (Millipore Sigma, Burlington, MA, USA). For this assay, the cells at a density 1 × 10^5^ were seeded in 6-well plates on porous scaffolds (scaffold-TGFβ1 and scaffold without peptides), as described in Section 2.6, and cultured for 21 days at 37 °C and 5% CO_2_. Then, the cells were rinsed with 1× PBS and fixed in 2.5% *w*/*v* glutaraldehyde solution at room temperature for 30 min. After fixation, the cells were incubated in a 1% *w*/*v* Alcian Blue solution pH 2.5 at room temperature for 30 min and rinsed with 0.1 M hydrochloric acid. The images were captured at 10× magnification using a Nikon DS-Fi3 microscope camera. The Alcian Blue staining was quantified by incubating the stained pellets in 4 M guanidine hydrochloride solution (Millipore Sigma, Burlington, MA, USA) overnight at 4 °C. The optical density (OD) was determined using a microplate reader (Biotek Plate Reader) at 600 nm [21].

### 2.10. SEM Analysis

Borosilicate glass coverslips were coated with scaffold-TGF1 into 24-well plates and sterilised as described in Section 2.6. Then, the cells were seeded on scaffolds at a density of 2 × 10^4^ cells per well in the presence of full α-MEM and incubated at 37 °C and 5% CO_2_. After 21 days, the cells were washed in PBS, fixed with 3% *w*/*v* glutaraldehyde (Millipore Sigma, Burlington, MA, USA) in 0.1 M sodium cacodylate containing 0.1 M sucrose at pH 7.4, and dehydrated with a graded series of increasing concentrations of ethanol and hexamethyldisilazane. Then, they were air-dried in the hood for 20–30 min before finally being carbon coated and observed under SEM (scanning electron microscopy) analysis (JEOL J.S.M. 840A, Tokyo, Japan), electronic microscopy at 20 kV, and Structural Characterization of Materials Laboratory of the Department of Physics at Aristotle University of Thessaloniki.

### 2.11. RNA Isolation and cDNA Synthesis

Total RNA was isolated from hDPSCs using the NucleoSpin RNA kit (Macherey-Nagel, Düren, Germany) according to the manufacturer’s instructions. A small amount of sample (2–3 μL) was diluted with sterile water (800 μL) and the absorbance of the solution was measured spectrophotometrically at 260 nm. The yield (from approximately 10^6^ cells) was 20 μg total RNA. The optical densities at the 260 nm and 280 nm (OD260/OD280) ratio were applied for assessing the purity of the samples. The ratio for all RNA samples was around 1.9, which indicates high purity according to the manufacturer’s guidelines.

The PrimeScript RT reagent kit (Takara, Kukatsu, Shiga, Japan) was utilized to generate cDNA. A total of 0.5 g RNA, 25 pmol Oligo-dt primer, 50 pmol random 6mers, 1× PrimeScript Buffer, and 0.5 μL PrimeScript enzyme mix were mixed together on ice and incubated at 37 °C for 15 min for each 20 μL reaction. After inactivating the reverse transcriptase for 5 s at 85 °C, the cDNA concentration was measured spectrophotometrically at 260 nm.

### 2.12. Real-Time PCR

The relative gene expression was quantified using a StepOne Real-time PCR System (ThermoFisher Scientific, Waltham, MA, USA) and KAPA SYBR FAST qPCR Kit Master Mix (2×) ABI PRISM, according to manufacturer’s directions. The expressions of all genes of interest were normalized against housekeeping genes *GAPDH* and *RPLPO*. Eurofins Genomics (Ebersberg, Germany) provided all the primers, and their sequences are presented in Table S5. The annealing temperature for all primers was 60 ± 1 °C.

### 2.13. Protein Extracts and Western Blotting Analysis

hDPSCs were seeded and grown on scaffolds for 21 days to perform immunoblotting experiments, as described in Section 2.6. The cells were then lysed, and the proteins GAPDH, Smad-2, phospho-Smad-2, Erk-1/2, and phospho-Erk-1/2 were identified in the protein extracts using the procedure employed by Mantsou et al. (2023) [17]. Briefly, protein extracts were collected after lysing the cells with RIPA Buffer containing a protease inhibitor cocktail, followed by centrifugation. In total, 50 µg of the total protein extracts were separated by SDS-polyacrylamide gel electrophoresis and transferred onto nitrocellulose membranes, which were subsequently blocked with skimmed milk solution in 1x PBS before being incubated in primary (1:1000) and secondary (1:2000) antibody solutions. The visualization of protein bands was accomplished by incubation of the membranes in 1× alkaline phosphatase buffer with substrate NBT and BCIP (Millipore Sigma, Burlington, MA, USA). Primary monoclonal antibodies for Erk1/2 (MAPK 42/44), phospho-Erk1/2 (phospho-MAPK 42/44), Smad-2, phospho-Smad-2, and secondary antibody (goat anti-rabbit IgG, alkaline phosphatase conjugated) were purchased from Cell Signaling Technology (Danvers, MA, USA). The primary polyclonal antibody for GAPDH was purchased from Santa Cruz Biotechnology (Dallas, TX, USA).

The ImageJ 1.53 software was used to quantify band intensities on the blots. The ratios phospho-Smad-2:Smad-2 and phospho-Erk1/2:Erk1/2 were visualized using the GraphPad Prism 8.2.1 software.

### 2.14. Statistical Analysis

The values on the graphs were shown as the mean standard ± standard deviation (SD) of experiments performed in triplicates (real-time PCR) and hexaplicates (MTT, BrdU). For unpaired samples, statistically significant differences were computed using Student’s *t*-tests, and differences were regarded statistically significant at the level *p* ≤ 0.05. GraphPad Prism 8.2.1 was used to create statistical analyses and graphics.

## 3. Results

### 3.1. Synthesis of Biomaterial with Elastin, Silk Fibroin, and Mussel-Foot Adhesive Protein Properties That Contains a Genetically Incorporated TGF-β1 Peptide

**Figure 1:**
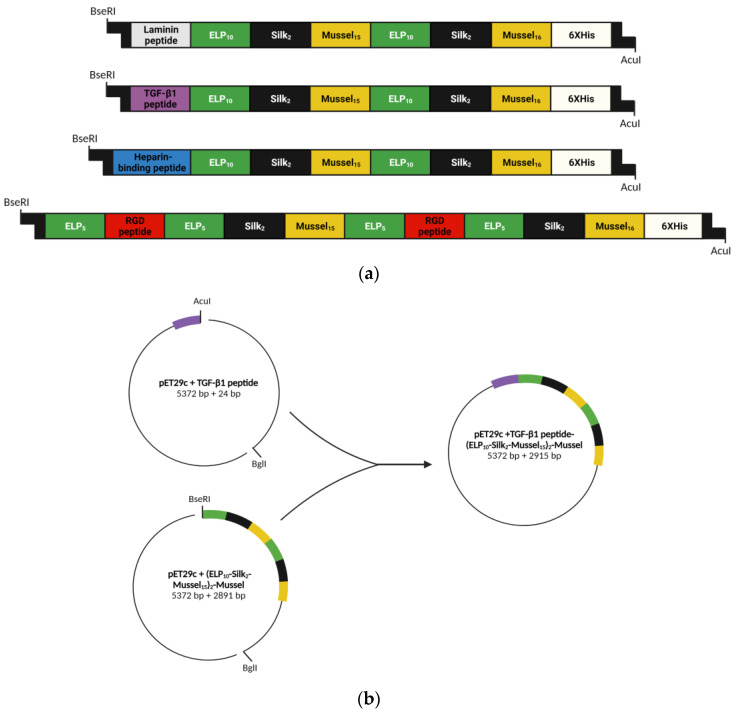
Schematic representations of (**a**) the structure of the synthesized genes which encode the polypeptides that constitute the scaffold for chondrogenesis, and (**b**) the assembly of the gene “TGF-β1 peptide-(ELP_10_-Silk_2_-Mussel_15_)_2_-Mussel-6xHis”. Colors in (**b**) correspond to building blocks shown in (**a**). The schematics were created with BioRender.com (accessed on 24 May 2023).

The gene sequence for a recombinant polypeptide was synthesized which combines DNA sequences inspired from human tropoelastin, silk fibroin, and mussel-foot adhesive protein-1 as well as an active peptide from the carboxyterminal region of TGF-β1, “YYVGRKPK”, which has been shown to promote the chondrogenic differentiation of human dental pulp stem cells (Pitou et al., 2023, submitted for publication) [18]. A coding sequence for a 6-histidine tag (6xHis tag) was included at the 3′ end of each gene, followed by two stop codons. Similar genes have been synthesized in our lab that contain, instead of the “TGF-β1 peptide” sequence, sequences for cell-attachment peptides from fibronectin or laminin a2 or a heparin-binding peptide (Figure 1a). The combination of the polypeptides encoded by these genes were the constituents of a novel scaffold for chondrogenesis.

The assembly of the complete gene “TGF-β1 peptide-(ELP_10_-Silk_2_-Mussel_15_)_2_-Mussel-6xHis” was performed with the PRe-RDL method [10]. The final recombinant plasmid was screened by double digestion with NdeI and XhoI (Figure S1b). A schematic representation of the gene assembly process is depicted in Figure 1b.

**Figure 2:**
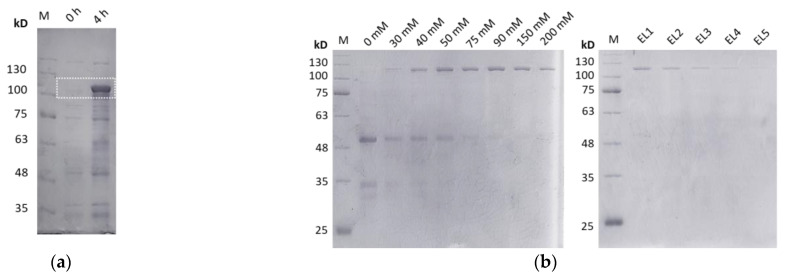
(**a**) Electrophoresis in 8% *w*/*v* SDS-polyacrylamide gel of cell extracts before and after overexpression of the polypeptide “TGF-β1 peptide-(ELP_10_-Silk_2_-Mussel_15_)_2_-Mussel-6xHis” (20 μL of cell extract per lane). The band that corresponds to the overexpressed polypeptide is shown in the box. 0 h: before induction of overexpression, 4 h: 4 h after induction of overexpression with 1 mM IPTG. (**b**) Electrophoresis in 10% *w*/*v* SDS-polyacrylamide gels of the fractions after purification of the polypeptide from BL21 *E. coli* cells (10 μg of protein per lane). A total of 0–200 mM washes with solutions containing increasing concentrations of imidazole (0–200 mM), EL1–EL5: elution with solution containing 250 mM imidazole. M: protein marker.

The gene was overexpressed in BL21 (DE3) *E. coli* bacterial cells. The predicted molecular mass of the polypeptide was ~90,094 Da and the corresponding band after SDS-polyacrylamide gel electrophoresis was observed slightly above the 100-kD protein marker, since, due to its fibrous structure, the electrophoretic mobility of the polypeptide differs from globular proteins (Figure 2a). The cells were lysed by sonication and the polypeptide was purified by Ni-NTA affinity chromatography under denaturing conditions (Figure 2b).

In our previous research, a scaffold was made by the crosslinking of polypeptides containing ELP–Silk–Mussel domains and either of the following peptides: a fibronectin peptide, a laminin peptide, or a heparin-binding peptide, resulting in a polymer network with cell-adhesive and biomimetic properties. The polypeptides were crosslinked with the use of hexamethylene diisocyanate (HDI). Using the same protocol in this project, a “scaffold-TGFβ1” was constructed by crosslinking of the aforementioned polypeptides with the polypeptide “TGF-β1 peptide-(ELP_10_-Silk_2_-Mussel_15_)_2_-Mussel-6xHis”, using HDI. The compositions of “scaffold-TGFβ1” and of a scaffold without the TGF-β1 peptide (scaffold without peptides) are described on Table 1.

**Figure 3:**
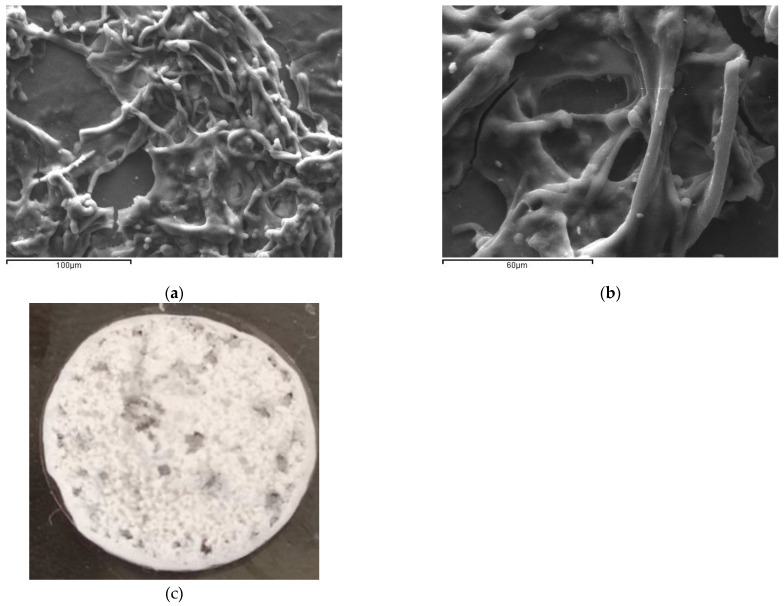
(**a**,**b**) Imaging of the micromorphology of the porous surface of scaffold-TGFβ1 with SEM at different magnifications. Scale bars of 100 μm and 60 μm are included. (**c**) Macroscopic picture of scaffold-TGFβ1 on borosilicate glass coverslip.

The crosslinked polymers were subjected to salt-leaching for the formation of porous scaffolds [19] (Figure 3). The micromorphology of the scaffold surface was observed with scanning electron microscopy (SEM), which revealed a highly porous fibrous network with interconnected pores (Figure 3a,b). Figure 3c depicts a macroscopic picture of the scaffold-TGFβ1.

### 3.2. Rheological Characterization

**Figure 4:**
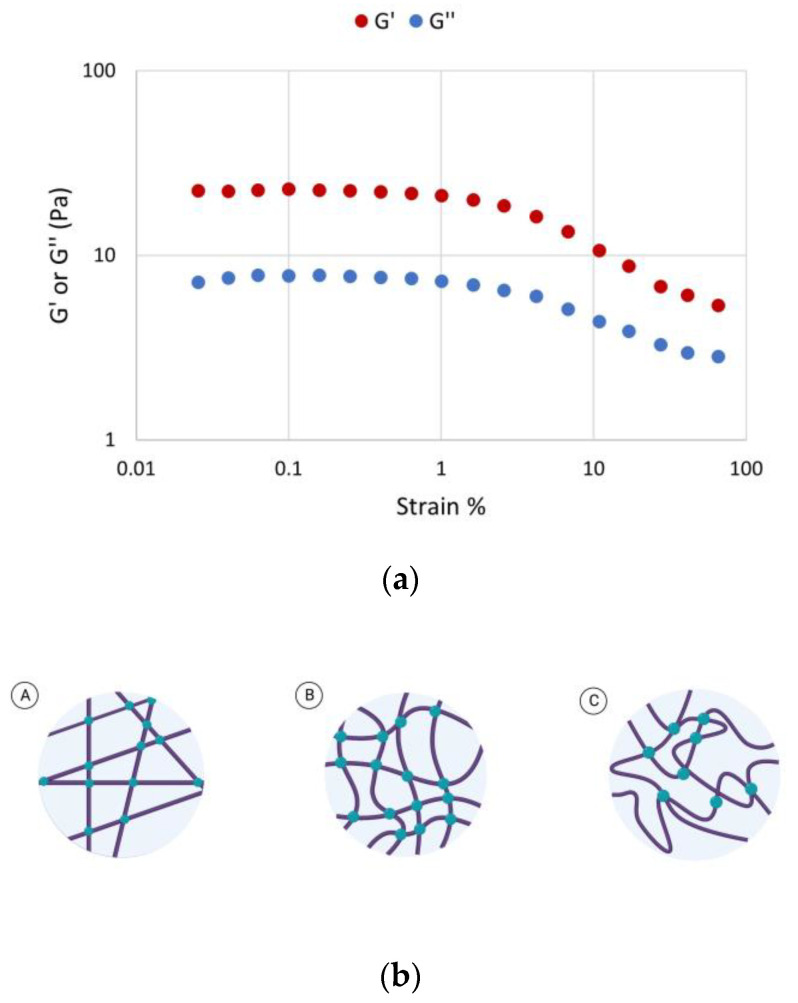
Identification of the LVR of the crosslinked scaffold-TGFβ1. (**a**) Plot of the elastic (G′ ± 2 Pa) and viscous modulus (G″ ± 0.2 Pa) as a function of strain (%) in the crosslinked biomaterial at 37 °C. (**b**) Schematic representation of the behavior of the biomaterial at 37 °C in DMEM. The crosslinked scaffold-TGFβ1 behaves as a semi-rigid (B), rather than a fully flexible (C) or a rigid (A), polymer. Lines show the protein fibers and dots show crosslinking sites. Created with BioRender.com (accessed on 24 May 2023). All data correspond to different aliquots of the same samples in order to avoid preshearing effects or different sample preparations in order to show reproducibility.

The rheological behavior of scaffold-TGFβ1 before and after the crosslinking, was characterized at 37 °C, at a concentration 6 mg/mL in DMEM. Firstly, the linear viscoelastic region of the crosslinked biomaterial (LVR) was determined [22]. For this purpose, strain sweeps were performed, which showed that the crosslinked scaffold-TGFβ1 formed a network at the crosslinked state, as shown by its elasticity to viscosity ratio (G′ > G″). The biomaterial also had a short LVR which extended up to maximum ~15% strain (Figure 4a), as it has been reported previously for other scaffolds that were synthesized in our lab [17]. This shows that the scaffold behaves more like a semi-rigid polymer network at the crosslinked state at 37 °C, rather than a fully rigid or flexible material (Figure 4b).

**Figure 5:**
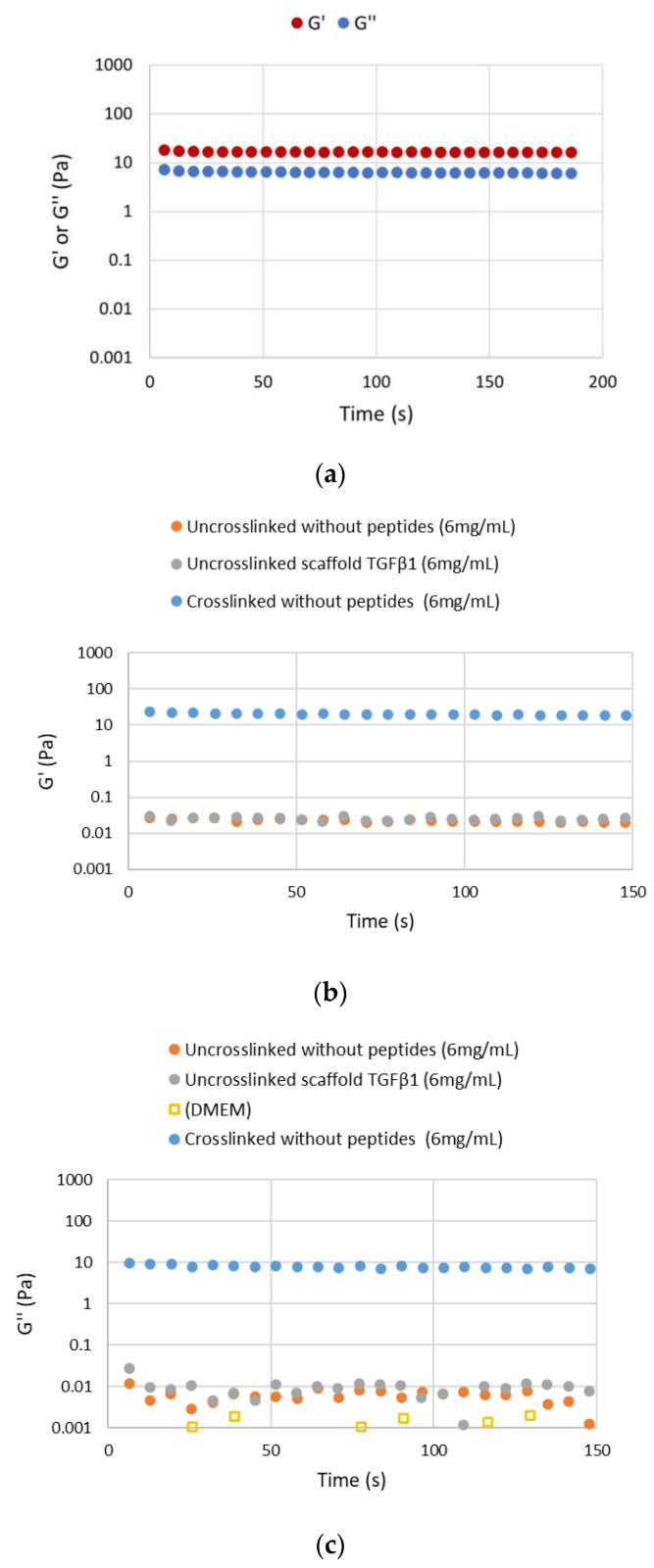
The viscoelastic properties of crosslinked and uncrosslinked biomaterials within the LVR (at 3% strain). (**a**) Plot of the elastic (G′ ± 0.2 Pa) and viscous modulus (G″ ± 0.2 Pa) of crosslinked scaffold-TGFβ1 (6 mg/mL) as a function of time (s) at 37 °C. (**b**) Plot of the elastic modulus (G′) of crosslinked and uncrosslinked scaffolds (6 mg/mL in DMEM) as a function of time (s) at 37 °C (blue set: G′ ± 0.2 Pa, orange and grey sets: G′ ± 0.002 Pa). (**c**) Plot of the viscous modulus (G″) of crosslinked and uncrosslinked scaffolds (6 mg/mL) and culture medium DMEM as a function of time (s) at 37 °C (blue set: G′ ± 0.2 Pa, orange and grey sets: G′ ± 0.004 Pa, yellow set: G′ ± 0.0005 Pa). All data correspond to different aliquots of the same samples in order to avoid preshearing effects or different sample preparations in order to show reproducibility.

Furthermore, time sweeps were performed with application of strain within the LVR (3% strain) for 150 s, on the crosslinked and uncrosslinked scaffold-TGFβ1 and scaffold without peptides. As shown in Figure 5, the G′ and G″ values of both biomaterials in the crosslinked state were higher by three orders of magnitude compared to the uncrosslinked state (~10 Pa vs. ~0.01 Pa). The viscous modulus (G″) of the uncrosslinked scaffolds was slightly higher than the culture medium (0.01 Pa and 0.001 Pa, respectively). Thus, it was verified that after crosslinking, the scaffolds formed extensive 3-dimensional networks in DMEM at 37 °C exhibiting typical viscoelastic behavior (G′ = 3.5 ± 1.0 × G″). These networks were similar in scaffolds with or without the TGF-β1 peptide and they were stable with time.

**Figure 6:**
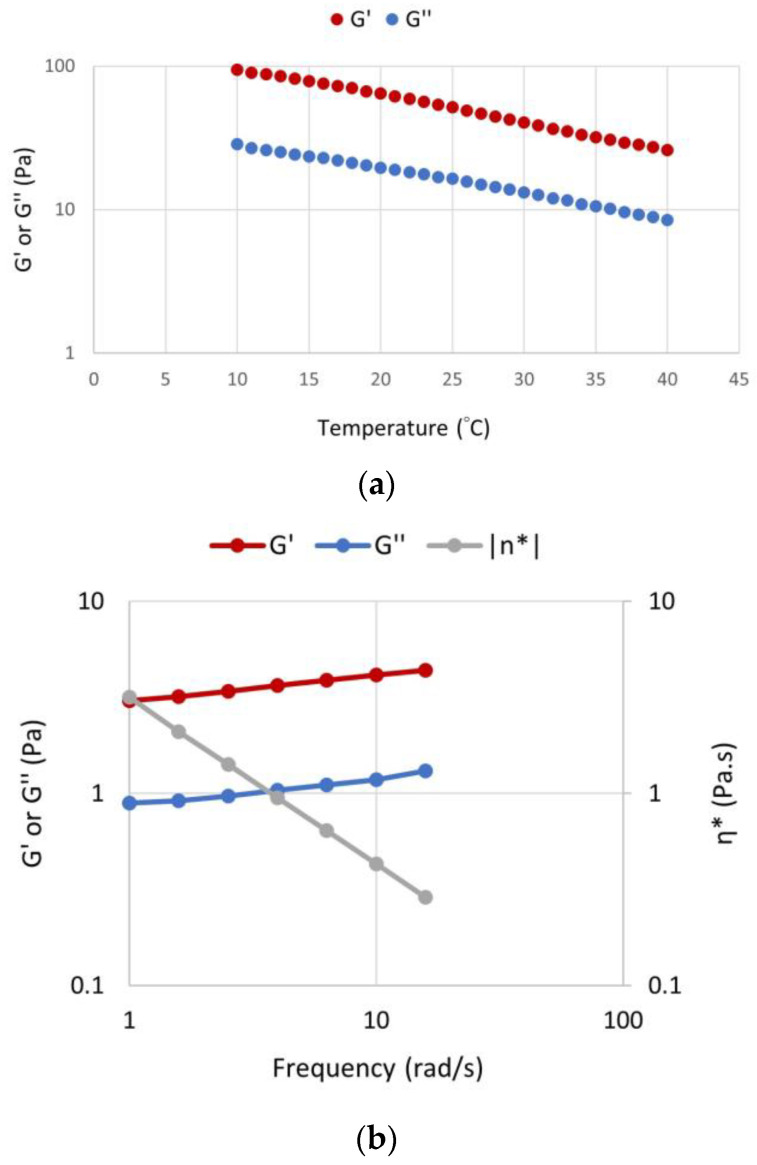
(**a**) Temperature sweep on the crosslinked scaffold-TGFβ1 at the range 10–40 °C. The viscoelastic properties of the crosslinked scaffold (G′ ± 0.2 Pa and G″ ± 0.2 Pa) showed a tendency to decrease with increasing temperature. (**b**) Frequency sweep on the crosslinked scaffold-TGFβ1 (6 mg/mL) at 37 °C (G′ ± 0.2 Pa and G″ ± 0.2 Pa). “η*”: dynamic viscosity. All data correspond to different aliquots of the same samples in order to avoid preshearing effects or different sample preparations in order to show reproducibility.

To determine the effect of temperature on the behavior of crosslinked scaffold-TGFβ1, the elastic and viscous moduli were measured at the range 10–40 °C (Figure 6a). By increasing the temperature, the materials exhibited a typical temperature dependence for polymer networks, i.e., by increasing the temperature, the magnitudes of viscoelastic parameters decreased gradually with at a rate 10 Pa/°C. This is consistent with our previous observations on the scaffold without peptides and a similar scaffold that contained a peptide for osteogenesis instead of a TGF-β1 peptide. A frequency sweep on the crosslinked scaffold-TGFβ1 (6 mg/mL) showed that the crossover angular frequency was relative low, well below 1 rad s^−1^, indicating that the network relaxation time is very long. This highlights the stable nature and long lifetime of the crosslink points and it agrees with what would be expected for a covalently crosslinked network (Figure 6b). The frequency value of 1 Hz (6.28 rad/s) was selected for the experiments.

**Figure 7:**
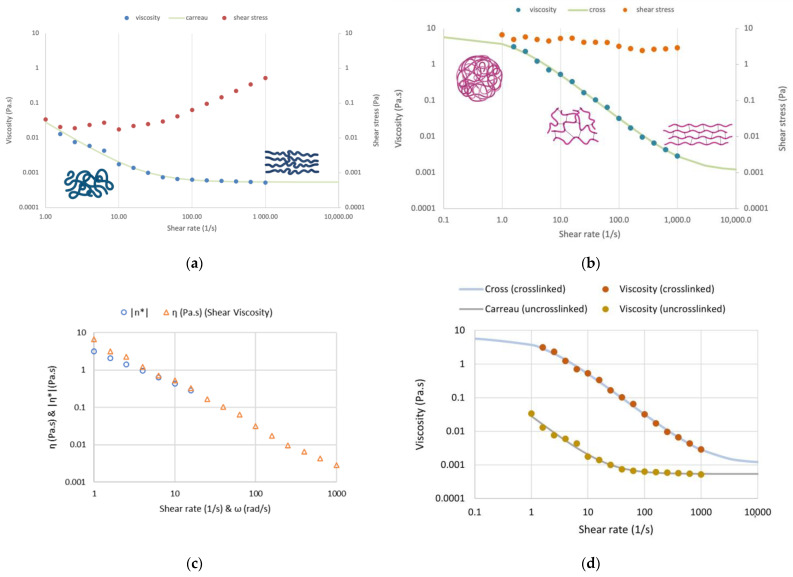
(**a**) Flow step on the uncrosslinked scaffold-TGFβ1 (6 mg/mL) at 37 °C. (**b**) Flow step on the crosslinked scaffold-TGFβ1 (6 mg/mL) at 37 °C. (**c**) Cox–Merz diagram of crosslinked scaffold-TGFβ1 (6 mg/mL) at 37 °C. (**d**) Flow step on the crosslinked compared to uncrosslinked scaffold-TFGβ1 (6 mg/mL) at 37 °C “η*”: dynamic viscosity. The standard deviation for dynamic viscosity was η* ± 0.0003 Pa·s. Schematics were made with BioRender.com (accessed on 24 May 2023).

Finally, flow measurements were performed on crosslinked and uncrosslinked scaffold-TGFβ1, which showed clearly that the crosslinked material had shear-thinning properties, as viscosity decreased with increasing shear rate (Figure 7). The crosslinked scaffold exhibited typical non-Newtonian behavior with strong shear-thinning which can be fitted well with the cross model (Figure 7b–d). This may be due to the alignment of the 3D network in the direction of flow; the stronger the shear field, the better the alignment. Even the uncrosslinked scaffold showed moderate non-Newtonian behavior due again to the alignment of the polypeptide chains to the shear field (Figure 7a,d).

### 3.3. Assessment of Cytotoxicity of the Scaffolds on hDPSCs and of Their Effect on Cell Proliferation

**Figure 8:**
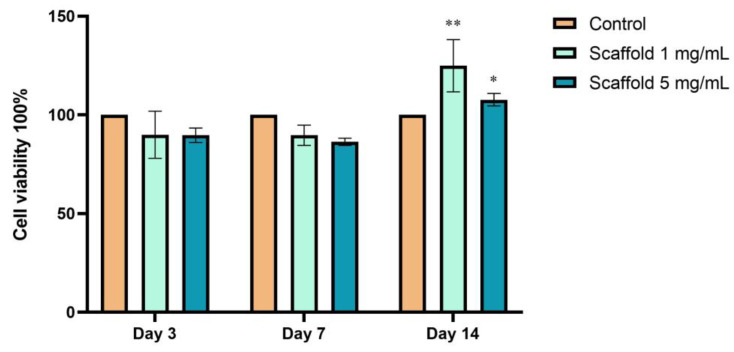
Evaluation of the cytotoxicity of scaffold-TGFβ1 to hDPSCs, by MTT assay. The assay was performed at 3, 7, and 14 days of culture. The OD was measured at 570 nm with a reference filter at 630 nm. “Control”: hDPSCs in full a-MEM. The data are presented as mean ± SD values of % cell viability. Asterisks (*) and (**) indicate statistically significant differences (*p* ≤ 0.05 and *p* ≤ 0.01, respectively) compared to the control cells.

The viability of hDPSCs on the scaffold-TGFβ1 was assessed by an MTT cytotoxicity assay (Figure 8). The cells were cultured on scaffold-TGFβ1, which had been applied on the surface of the wells of a 96-well plate at two concentrations, 1 mg/mL or 5 mg/mL, for up to 14 days. The MTT assay showed that the viability of cells on both concentrations of the scaffold was similar to control cells after 3 and 7 days of culture and increased after 14 days of culture. Specifically, on the 14th day, the viability of hDPSCs on 1 mg/mL scaffold-TGFβ1 was ~25% higher than the control cells (*p* < 0.01), verifying that the scaffold is non-toxic to the cells. The concentration of 1 mg/mL was selected for investigation of the ability of the scaffold to induce the chondrogenic differentiation of hDPSCs.

**Figure 9:**
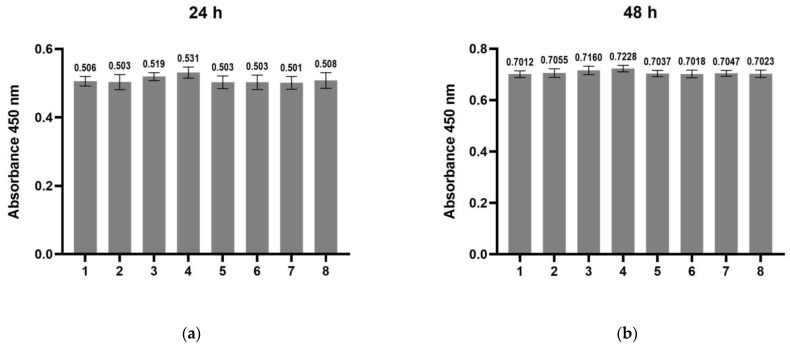

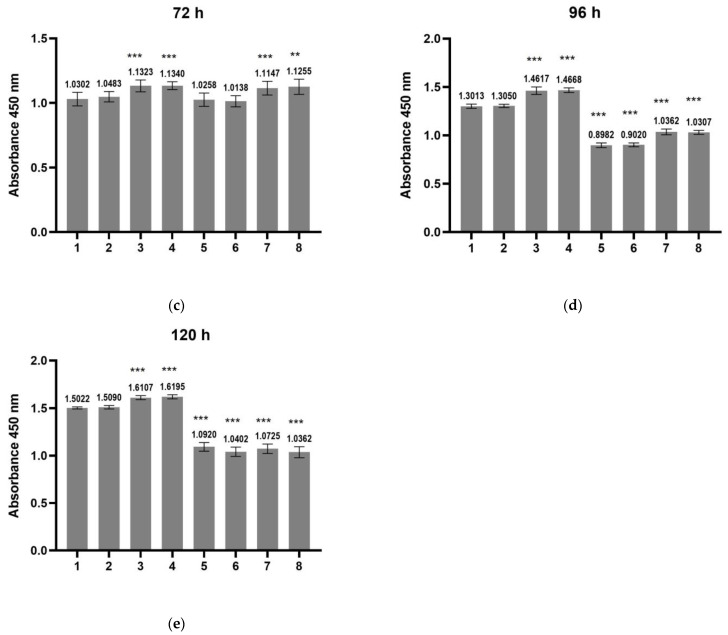
Evaluation of the proliferation of hDPSCs cultured on scaffold-TGFβ1 or scaffold without peptides or of hDPSCs treated with TGF-β1 peptide by BrdU assay. The assay was performed at 24 h (**a**), 48 h (**b**), 72 h (**c**), 96 h (**d**), and 120 h (**e**) of culture. (1) Control cells (hDPSCs cultured in complete α-ΜΕΜ), (2) hDPSCs cultured in complete α-ΜΕΜ and treated with 10 ng/mL TGF-β1 peptide, (3) hDPSCs cultured on scaffold without peptides in complete α-ΜΕΜ, (4) hDPSCs cultured on scaffold-TGFβ1 in complete α-ΜΕΜ, (5) hDPSCs cultured in chondrogenesis medium, (6) hDPSCs cultured in chondrogenesis and treated with 10 ng/mL TGF-β1 peptide, (7) hDPSCs cultured on scaffold without peptides in chondrogenesis medium, and (8) hDPSCs cultured on scaffold-TGFβ1 in chondrogenesis medium. The optical density (OD) was measured against a blank (cell-free and BrdU-free wells) at 450 nm with a reference filter at 690 nm. The data are presented as mean ± SD values of absorbance at 450 nm. Asterisks (**) and (***) indicate statistically significant differences (*p* ≤ 0.01 and *p* ≤ 0.001, respectively) compared to control cells.

The effect of scaffold-TGFβ1, scaffold without peptides, or TGF-β1 peptide alone on cell proliferation was assessed by BrdU assay after 24–120 h of culture (Figure 9). The following samples were used: control cells, cells in chondrogenesis medium, cells treated with 10 ng/mL TGF-β1 peptide (in a-MEM or in chondrogenesis medium), cells cultured on scaffold without peptides (in a-MEM or in chondrogenesis medium), and cells cultured on scaffold-TGFβ1 (in a-MEM or in chondrogenesis medium). The proliferation rate of all the treated cells was similar to the control cells at 24 h and 48 (Figure 9a,b), but a slight increase (10–15%) was observed after 72 h in cells that had been cultured on the scaffolds compared to the control cells, both in α-ΜΕΜ and in chondrogenesis medium (*p* < 0.01). After 96 h, the proliferation rate decreased in all cell groups that had been cultured in chondrogenesis medium due the initiation of differentiation (Figure 9d,e).

### 3.4. A Scaffold That Contains the Active TGF-β1 Peptide “ΥΥVGRKPK” Induces Chondrogenesis in hDPSCs

To evaluate the capacity of scaffold-TGFβ1 to induce the chondrogenic differentiation of stem cells, hDPSCs were cultured on 1 mg/mL scaffold-TGFβ1 for 21 days. For comparison, cells were also cultured on 1 mg/mL scaffold without peptides (scaffold that does not contain growth factor peptides) or they were treated with exogenous TGF-β1 and cultured for 21 days, while control cells were cultured without any scaffold. Then, the differentiation was evaluated by quantification of the expression of chondrogenic gene markers by investigation of chondrogenic signaling (Smad-2 and phospho-Erk1/2 pathways) and by staining of ECM glycosaminoglycans (Alcian Blue staining). Eight samples were used for the real-time PCR experiments: (1) Control cells (hDPSCs cultured in complete α-ΜΕΜ), (2) hDPSCs cultured in chondrogenesis medium, (3) hDPSCs cultured in complete α-ΜΕΜ and treated with 10 ng/mL TGF-β1 peptide, (4) hDPSCs cultured in chondrogenesis and treated with 10 ng/mL TGF-β1 peptide, (5) hDPSCs cultured on scaffold without peptides in complete α-ΜΕΜ, (6) hDPSCs cultured on scaffold without peptides in chondrogenesis medium, (7) hDPSCs cultured on scaffold-TGFβ1 in complete α-ΜΕΜ, and (8) hDPSCs cultured on scaffold-TGFβ1 in chondrogenesis medium.

**Figure 10:**
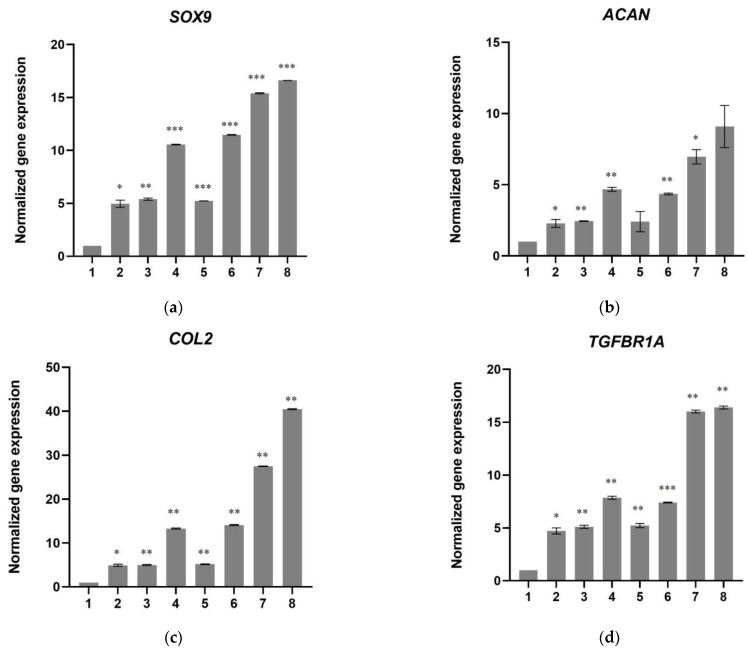

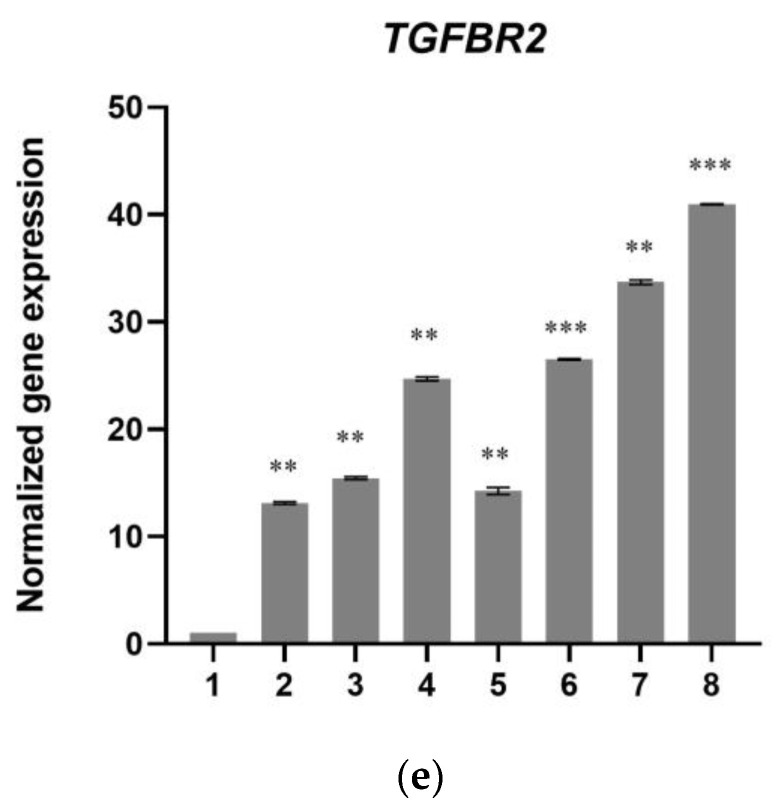
Relative quantification of the mRNA levels of chondrogenesis markers (**a**) *SOX9*, (**b**) *ACAN*, (**c**) *COL2A1*, (**d**) *TGFBR1A,* and (**e**) *TGFBRR2* after 21 days of differentiation of hDPSCs on the scaffolds and without scaffold. (1) Control cells, (2) cells in chondrogenesis medium, (3) cells treated with 10 ng/mL TGF-β1 peptide (in α-MEM), (4) cells treated with 10 ng/mL TGF-β1 peptide (in chondrogenesis medium), (5) cells on scaffold without peptides (in α-ΜΕΜ), (6) cells on scaffold without peptides (in chondrogenesis), (7) cells on scaffold-TGFβ1 (in α-ΜΕΜ), and (8) cells on scaffold-TGFβ1 (in chondrogenesis medium). The normalization of Ct values was performed against two housekeeping genes, *GAPDH* and *RPLPO*. The data are presented as the mean ± SD values (*n* = 3). Asterisks (*), (**), and (***) indicate statistically significant differences (*p* ≤ 0.05, *p* ≤ 0.01 and *p* ≤ 0.001, respectively) compared to control cells.

After 21 days of culture, total RNA was isolated from the cells for analysis. As shown in Figure 10, all chondrogenesis marker genes (*SOX9*, *COL2*, *ACAN*, *TGFBR1A,* and *TGFBR2*) had the highest expression in cells that had been cultured on scaffold-TGFβ1. In more detail, cells in chondrogenesis medium had 2 times higher aggrecan (*ACAN*); 5 times higher *COL2*, *SOX9,* and *TGFBR1A;* and 13 times higher *TGFBR2* expression than control cells. Gene expression levels were similar in cells cultured on scaffold without peptides in α-ΜΕΜ, but 2–3 times higher when the cells were cultured on the same scaffold in chondrogenesis medium. In cells that were treated with TGF-β1 peptide, the expression levels of genes *ACAN*, *COL2*, *SOX9, TGFBR1A,* and *TGFBR2* were similar to those observed in cells cultured on scaffold without peptides. However, in cells that had been cultured on scaffold-TGFβ1, *ACAN* expression was 7–9 times higher than in the control cells, *SOX9* expression was 15–16 times higher, *TGFBR1A* 16 times, *COL2* 27–40 times, and *TGFBR2* 34–40 times higher than in the control hDPSCs.

**Figure 11:**
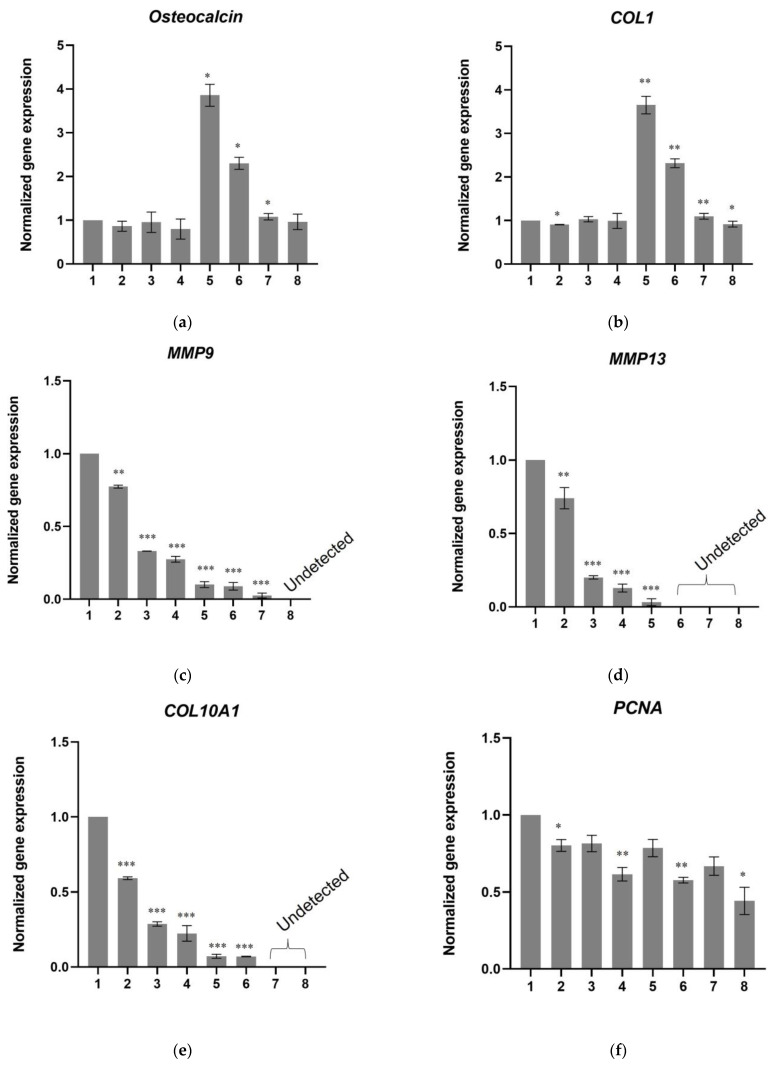

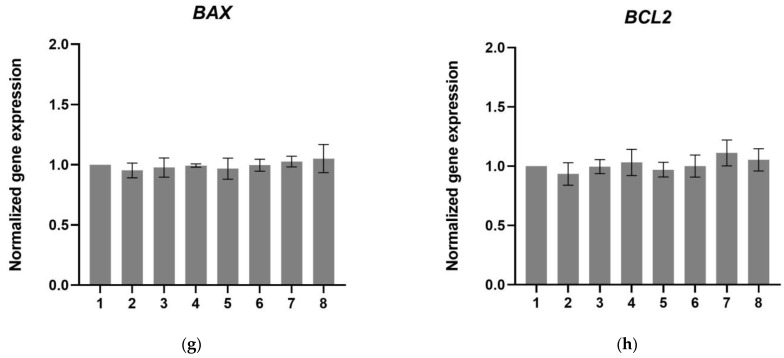
Relative quantification of the mRNA levels of (**a**) *Osteocalcin*, (**b**) *COL1A1*, (**c**) *MMP9*, (**d**) *MMP13*, (**e**) *COL10A1*, (**f**) *PCNA*, (**g**) *BAX,* and (**h**) *BCL2* after 21 days of differentiation of hDPSCs on the scaffolds and without scaffold. (1) Control cells, (2) cells in chondrogenesis medium, (3) cells treated with 10 ng/mL TGF-β1 peptide (in α-MEM), (4) cells treated with 10 ng/mL TGF-β1 peptide (in chondrogenesis medium), (5) cells on scaffold without peptides (in α-ΜΕΜ), (6) cells on scaffold without peptides (in chondrogenesis), (7) cells on scaffold-TGFβ1 (in α-ΜΕΜ), and (8) cells on scaffold-TGFβ1 (in chondrogenesis medium). The normalization of Ct values was performed against two housekeeping genes, *GAPDH* and *RPLPO*. The data are presented as the mean ± SD values (*n* = 3). Asterisks (*), (**) and (***) indicate statistically significant differences (*p* ≤ 0.05, *p* ≤ 0.01 and *p* ≤ 0.001, respectively) compared to control cells. No statistically significant differences were observed between samples in *BAX* and *BCL2* gene expression. *MMP9*, *MMP13,* and *COL10A1* were detected after Ct = 37, while *IL1b* and *TNFa* mRNA levels were undetected in all samples.

In parallel, the mRNA levels of genes for collagen type I alpha 1 chain (*COL1*), osteocalcin, metalloproteases MMP9 and MMP13, and collagen type X alpha 1 chain (*COL10A1*) were quantified after 21 days of culture. As shown in Figure 11a–e, the expression of *COL2* and *Osteocalcin* was at similar levels (or in some cases lower) in cells treated with TFG-β1 peptide or in cells cultured on scaffold-TGFβ1 as in control cells. An upregulation of these genes was observed in cells cultured on scaffold without peptides, which is consistent with previously published results that showed this scaffold can induce osteogenesis at a certain degree [17]. *MMP9*, *MMP13,* and *COL10A1* mRNA levels were detected in most samples after the 37^th^ cycle, while they were completely undetected in the scaffold-TGFβ1 samples in our qPCR experiments.

Moreover, mRNA levels of *PCNA*, *BAX*, *BCL2*, *TNFa,* and *IL1b* were quantified by real-time PCR to investigate effects on cell proliferation, apoptosis, and inflammation. The expression of *PCNA* was decreased by approximately 20% in cells treated with TGF-β1 peptide and in cells cultured on scaffold without peptides and by approximately 35% in cells cultured on scaffold-TGFβ1 (in α-ΜΕΜ) compared to control cells (Figure 11f). This decrease, which was stronger where chondrogenesis medium had been added, is consistent with the evidence that shows the cells had differentiated into chondroblasts at 21 days. The expression levels of pro-apoptotic *BAX* and anti-apoptotic *BCL2* were found to be similar between all samples (Figure 11g,h). *IL1b* and *TNFa* mRNA levels were undetected in all samples.

**Figure 12:**
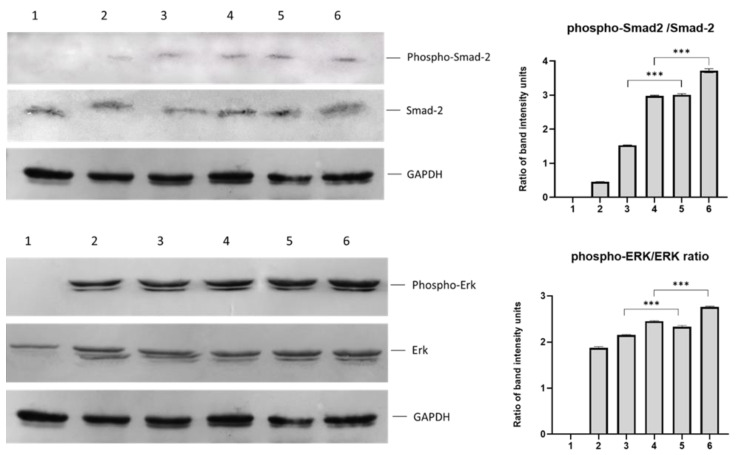
Western blotting against phospho-Smad-2, Smad-2, phospho-Erk1/2, Erk1/2, and GAPDH in protein extracts after 21 days of culture of hDPSCs on the scaffolds and without scaffold. (1) Control cells, (2) cells in chondrogenesis medium, (3) cells on scaffold without peptides (in α-ΜΕΜ), (4) cells on scaffold without peptides (in chondrogenesis), (5) cells on scaffold-TGFβ1 (in α-ΜΕΜ), and (6) cells on scaffold-TGFβ1 (in chondrogenesis medium). The bar charts depict phospho-Erk/Erk and phospho-Smad-2/Smad-2 ratios after quantification of band intensities in the blots, using the ImageJ 1.53t software. The data are presented as the mean ± SD values (*n* = 3). Asterisks (***) indicate statistically significant differences (*p* ≤ 0.001) compared to control cells.

For Western blotting and Alcian Blue staining assays, the following samples were used: (1) control cells (hDPSCs cultured in complete α-ΜΕΜ), (2) hDPSCs cultured in chondrogenesis medium, (3) hDPSCs cultured on scaffold without peptides in complete α-ΜΕΜ, (4) hDPSCs cultured on scaffold without peptides in chondrogenesis medium, (5) hDPSCs cultured on scaffold-TGFβ1 in complete α-ΜΕΜ, and (6) hDPSCs cultured on scaffold-TGFβ1 in chondrogenesis medium. Intracellular levels of phosphor-Erk1/2, Erk1/2, phosphor-Smad-2, and Smad-2 were detected by Western blotting (Figure 12). Phosphorylation of Erk-1/2 and Smad-2 was higher when cells were cultured on the scaffold-TGFβ1 compared to cells cultured on the scaffold without peptides and to cells without scaffold. This was also confirmed by calculating the phospho-Erk/Erk and phospho-Smad-2/Smad-2 ratios. Specifically, the phospho-Erk/Erk ratio in cells cultured on the scaffold-TGFβ1 was 22% (α-ΜΕΜ) and 45% (chondrogenesis medium) higher compared to cells in chondrogenesis medium without scaffold. Similarly, the phospho-Smad-2/Smad-2 ratio in cells cultured on scaffold-TGFβ1 was 92% (in α-ΜΕΜ) 139% (chondrogenesis medium) higher compared to cells in chondrogenesis medium without scaffold. No phosphorylated Erk1/2 or Smad-2 were detected in the control cells. These results demonstrated the efficient induction of Smad-2 and Erk-1/2 signaling pathways involved in chondrogenesis.

**Figure 13:**
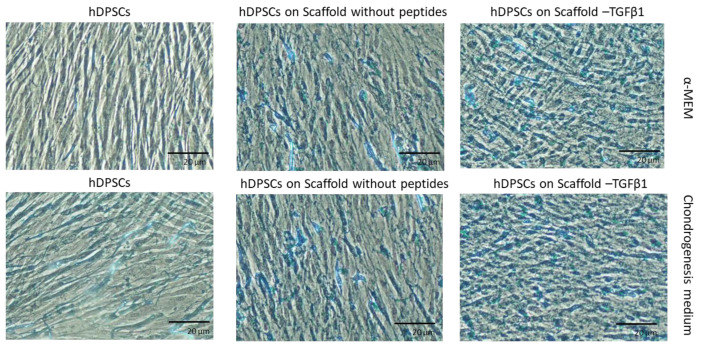
Detection of glycosaminoglycans in the extracellular matrix by Alcian Blue staining after 21 days of culture of hDPSCs on scaffolds and without scaffolds. The photographs were taken at 10× magnification with a Nikon DS-Fi3 microscope camera. The 20-μm scale bars are included in all photographs.

The deposition of glycosaminoglycans in the ECM was evaluated by Alcian Blue staining after 21 days of culture of hDPSCs on the scaffolds. As shown in Figure 13, the stained areas were abundant in hDPSCs cultured on scaffold without peptides, but the most abundant were in hDPSCs cultured on scaffold-TGFβ1. The results were also verified by semi-quantification of Alcian Blue staining (Figure S2, Supplementary Material).

**Figure 14:**
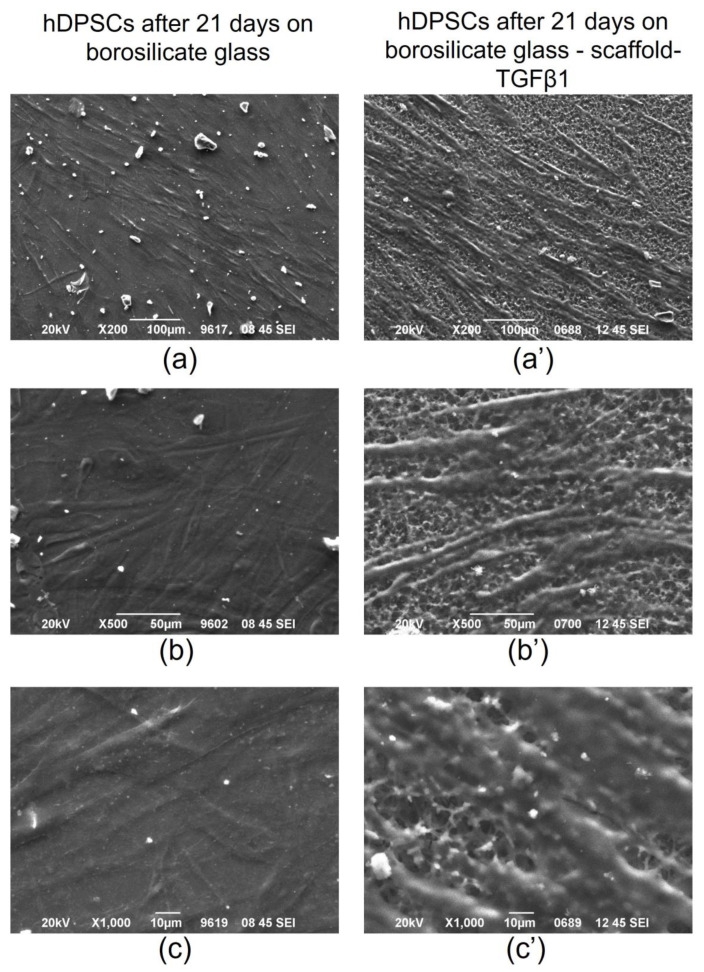
SEM images at (**a**) and (**a’**) magnification 200×: (**a**) hDPSCs cells on borosilicate glass without scaffold-TGFβ1 after 21 days, (**a’**): hDPSCs cells on scaffold-TGFβ1 onto borosilicate glass after 21 days, (**b**) and (**b’**) magnification 500×: (**b**) hDPSCs cells on borosilicate glass without scaffold-TGFβ1 after 21 days, (**b’**): hDPSCs cells on scaffold-TGFβ1 onto borosilicate glass after 21 days and (**c**) and (**c’**) magnification 1000×: (**c**) hDPSCs cells on borosilicate glass without scaffold-TGFβ1 after 21 days, (**c’**): hDPSCs cells on scaffold-TGFβ1 onto borosilicate glass after 21 days. The porosity of scaffold-TGFβ1 structure is clearly shown in the images (**a’**,**b’**,**c’**).

Furthermore, hDPSCs were seeded on scaffold-TGFβ1, which had been placed on borosilicate glass coverslips, and they were cultured for 21 days. Control hDPSCs were also cultured for 21 days on the borosilicate coverslips. Then, the cells were observed with scanning electron microscopy. As shown on Figure 14, the scaffold seems to provide a favorable environment for the cells.

## 4. Discussion

Cartilage engineering remains an issue to this day as tissue has low inherent capacity to heal and regenerate, while diseases that stem from its degeneration, such as osteoarthritis, are becoming more common [1,3]. To address this issue, we designed and produced an innovative biomaterial that contained amino acid sequences for cell adhesion and a TGFβ1 peptide for the induction of chondrogenic differentiation. This biomaterial is a network of crosslinked protein fibers, each of which contains multiple peptide repetitions from tropoelastin, silk fibroin, and MFP-1. Such protein domains are especially popular in biomedical engineering due to their exceptional mechanical characteristics, biodegradability, cytocompatibility, and low inflammatory reaction [8,13,16,23,24,25,26,27]. The embedding of integrin-binding sequences, such as “YAVTGRGDSPASSG” from fibronectin, enhances cell attachment and proliferation on biomaterials [8,25], while the sequence “YHYVTITLDLQQ” from laminin A2 has also been shown to bind effectively to the cell surface [28]. These peptides were inserted genetically in different fibers (polypeptides) of our biomaterial, as well as a peptide with strong ability to bind to heparin, “YPTQRARYQWVRCNP” [29], to increase binding of the biomaterial with the cell surface and with ECM proteoglycans. Finally, certain fibers displayed the TGF-β1 peptide “YYVGRKPK” at their N’-terminal end. These polypeptides were crosslinked to create a polymer network, “scaffold-TGFβ1”, that contained the combined properties.

Rheological measurements showed that the scaffold formed a viscoelastic three-dimensional network at 37 °C, stable with time and similar to the networks that had been described for similar scaffolds in our previous work [17]. The “scaffold-TGFβ1” behaves as a semi-rigid network at the crosslinked state, with temperature dependence typical of most polymers, and typical non-Newtonian behavior with strong shear-thinning properties, indicating the better alignment of the network in the direction of flow as the shear field increased, which is an important property for an injectable biomaterial. Even in the uncrosslinked state, scaffold-TGFβ1 showed moderate non-Newtonian behavior, indicating loose network formation. The crosslinked scaffold showed highly improved viscoelastic properties compared to the uncrosslinked and, therefore, was selected for further investigation.

MTT assays showed that the scaffold was non-toxic to hPDSCs. Moreover, BrdU assays showed that the proliferation rate of cells that had been cultured on the scaffolds was higher compared to control cells until 120 h of culture. Thus, it is proven that the scaffold provides favorable conditions for the cells.

Articular cartilage formation in vivo is regulated primarily by TGF-β1, whose signaling regulates the accumulation of mesenchymal stem cells and the induction of their differentiation to chondroblasts [30,31]. TGF-β1 binds to the heteromeric TβRII/ALK5 receptor to activate signaling cascades that ultimately result in the expression of transcription factor SOX9 at a higher expectable level. SOX9 leads to the increased production of collagen type II and aggrecan [32,33]. In the canonical pathway of TGF-β1, activated upon binding of the factor to receptor complex ALK5/TβRII, intracellular Smad-2/3 proteins are phosphorylated and then form a transcriptional regulatory complex with co-Smad (Smad-4) that induces chondrogenesis genes [34]. Through ALK5/TβRII, TGF-β1 also activates non-canonical pathways that involve MAP kinases, leading to increased expression of chondrogenesis genes [34].

To investigate the capacity of scaffold-TGFβ1 to promote chondrogenic differentiation, the mRNA levels of genes *SOX9* (SRY-box transcription factor 9), *COL2* (collagen type II alpha 1 chain), *ACAN* (aggrecan), *TGFBR1A* (ALK5 receptor), and *TGFBR2* (TβRII receptor) was quantified by real-time PCR in cells cultured on scaffold-TGFβ1 for 21 days and compared to cells cultured on scaffold without peptides, to cells cultured without scaffold but treated with TGF-β1 peptide, and to untreated cells. Our results showed that the scaffold-TGFβ1, even without chondrogenesis medium, enhanced the expression of the marker genes with high efficiency. The TGF-β1 peptide, when added in the α-ΜΕΜ culture medium, increased the expression of the chondrogenic marker genes to a similar degree to the scaffold without peptides. Chondrogenesis medium resulted in a further increase in gene expression in both cases, as it was expected.

Proliferating Cell Nuclear Antigen (PCNA) is a protein that is vital for the recruitment and function of DNA polymerase δ in eukaryotic cells, therefore, the expression of its gene (*PCNA*) indicates the rate of cell proliferation [35,36]. The decrease in mRNA levels of *PCNA* that were observed in cells treated with TGFβ1 and in cells cultured on scaffold without peptides or on scaffold-TGFβ1 compared to control cells after 21 days, are in agreement with our results showing that the cells had differentiated and, therefore, had exited the cell cycle. Neither the TGFβ1 peptide nor the scaffolds induced apoptosis, as evidenced by the expression levels of *BAX* and *BCL2* genes which were similar between the treated samples and the control. mRNAs of proinflammatory cytokines IL-1b 9 and TNFa were also not detected in any of the samples.

Immunoblotting was used to detect phosphorylated and total Smad-2 and Erk-1/2 in protein extracts. The phospho-Erk to Erk and phospho-Smad-2 to Smad-2 ratios revealed that scaffold-TGF1 triggered both pathways with high effectiveness, even in the absence of chondrogenesis medium. More specifically, the phospho-Smad-2 to Smad-2 ratio in cells cultured on scaffold without peptides in α-MEM was higher than in cells cultured in chondrogenesis medium without scaffolds, demonstrating that it can promote canonical Smad signaling to an extent. This impact was increased when cells were grown on scaffold-TGF1 in α-MΕΜ (*p* < 0.001). The utilization of chondrogenesis medium, which includes differentiation factors, boosted Smad-2 phosphorylation in both scaffolds. When cells cultured on scaffold without peptides were compared to cells cultured in chondrogenesis medium without scaffolds, the phospho-Erk to Erk ratio was slightly greater. The ratio increased when cells were cultivated on scaffold TGF-1 in α-MΕΜ, with a statistically significant difference (*p* ≤ 0.001), revealing that the scaffold-TGF-1 stimulated the Erk-1/2 pathway. The presence of chondrogenesis medium increased Erk-1/2 phosphorylation in both scaffolds.

Finally, staining of ECM glycosaminoglycans with Alcian Blue in hDPSCs after 21 days of culture on the scaffolds showed more abundant and intensively stained areas in the case of scaffold-TGFβ1.

SEM imaging of hDPSCs cultured on scaffold-TGFβ1 for 21 days showed that the scaffold provided a favorable environment for the cells.

**Figure 15:**
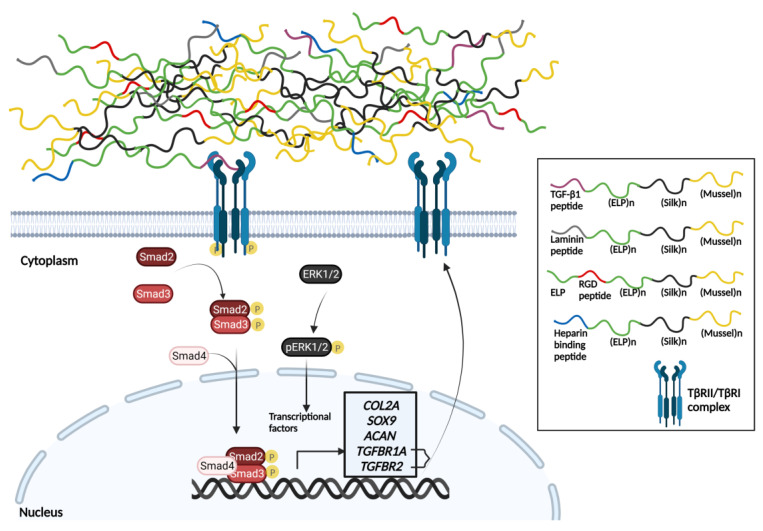
Schematic representation of the signal transduction induced by the scaffold-TGFβ1 in human dental pulp stem cells. Upon binding of the TGF-β1 peptide to the ΤβRII/TβRI receptor complex, the activated TβRI receptor phosphorylates and activates intracellular Smad-2/3 and Erk-1/2. Activated Smad-2/3 forms a complex with co-Smad (Smad-4), which translocates to the nucleus and acts as transcriptional activator of chondrogenesis-inducing transcription factors, such as SOX9. Similarly, the phosphorylated Erk-1/2 translocates to the nucleus and activates transcriptional regulators of chondrogenesis genes. Eventually, both signaling pathways enhance the production of proteins involved in the formation of cartilage extracellular matrix (collagen type II -*COL2*- and aggrecan -*ACAN*-), as well as type ΤβRI (TGFBR1A) and ΤβRII (*TGFBR2*). Created with BioRender.com (accessed on 24 May 2023).

These data strongly suggest that scaffold-TGF1 can activate chondrogenic signaling, resulting in increased expression of genes *SOX9*, *ACAN*, *COL2*, *TGFBR1A*, and *TGFBR2*, as well as the activation of chondrogenic differentiation and cartilaginous ECM formation (Figure 15). At the same time, it was verified that the scaffold does not induce hypertrophy in chondrocytes, as it was evidenced by the undetectable expression levels of *MMP9*, *MMP13*, and *COL10A1*, nor does it activate apoptotic or inflammatory processes.

## 5. Conclusions

Within this paper, we described the synthesis and thorough investigation of a “smart biomaterial” with a genetically incorporated TGF-β1 peptide for the delivery of chondrogenesis signals to hDPSCs. This biomaterial fulfils the safety requirements of a biomaterial for tissue regeneration, since it is composed entirely of peptide sequences from natural fibrous proteins that are non-toxic and non-immunogenic to the human body and has the necessary mechanical properties to support cartilaginous tissue formation. Considering the low capacity of cartilage to regenerate, we have synthesized a scaffold that enhances TGF-β1 signaling significantly, leading to the differentiation of stem cells into chondroblasts and the formation of hyaline cartilage ECM. TGF-β1 is a dimeric growth factor whose conformation is critical for its activity, but often difficult to maintain during its preparation. This disadvantage can be eliminated by using a more stable, short peptide (TGF-1 peptide) that has been genetically included in our scaffold.

## 6. Future Prospects

Currently running preliminary trials in dogs with knee osteoarthritis have shown that the scaffold-TGFβ1 injected with dog adipose-derived mesenchymal stem cells at the site induced the formation of cartilage and improved the clinical picture of the animals (unpublished results). The use of the scaffold-TGFβ1 as an injectable material with a patient’s autologous stem cells is proposed as a safe future alternative for the regeneration of cartilage and the permanent treatment of degenerative conditions, such as OA, and injuries.

## Figures and Tables

**Table 1:** Composition of the crosslinked scaffolds (“+” and “-“ signs show that the polypeptide is or is not contained in the respective scaffold.

Polypeptides	Scaffold-TGFβ1	Scaffold without Peptides
TGF-β1 peptide-(ELP_10_-Silk_2_-Mussel_15_)_2_-Mussel-6xHis	+	-
(ELP_5_-RGD peptide-ELP_5_-Silk_2_-Mussel_15_)_2_-Mussel-6xHis	+	+
Laminin peptide-(ELP_10_-Silk_2_-Mussel_15_)_2_-Mussel-6xHis	+	+
Heparin-binding peptide-(ELP_10_-Silk_2_-Mussel_15_)_2_-Mussel-6xHis	+	+

## Data Availability

The data presented in this study are available on request from the corresponding author.

## Supplementary Materials

The following supporting information can be downloaded at: https://www.mdpi.com/article/10.3390/biomedicines11071890/s1. Figure S1: Synthesis of the gene “TGF-β1 peptide-(ELP10-Silk2-Mussel15)2-Mussel-6xHis”; Figure S2: Quantification of Alcian Blue staining at 21 days; Table S1: Nucleotide and amino acid sequences of the building blocks; Table S2: Primer sequences for the synthesis of the DNA sequence encoding the “TGF-β1 peptide” by PCR; Table S3: Composition of the PCR reaction for the synthesis of the DNA sequence encoding the “TGF-β1 peptide”; Table S4: Conditions of the PCR reaction for the synthesis of the DNA sequence encoding the “TGF-β1 peptide”; Table S5: Primer sequences for real-time PCR; DNA and amino acid sequences for [TGF-β1 peptide-(ELP10-Silk2-Mussel15)2-Mussel-6xHis].
