# Supplementary material for: Fabrication of a Smart Fibrous Biomaterial That Harbors an Active TGF-β1 Peptide: A Promising Approach for Cartilage Regeneration
Source: Biomedicines. 2023 Jul 3;11(7):1890. doi: 10.3390/biomedicines11071890 (PMC10377373; doi:10.3390/biomedicines11071890)
Supplement: Supplementary file 1 [file biomedicines-11-01890-s001.zip › biomedicines-2443159-supplementary.pdf]

Figures

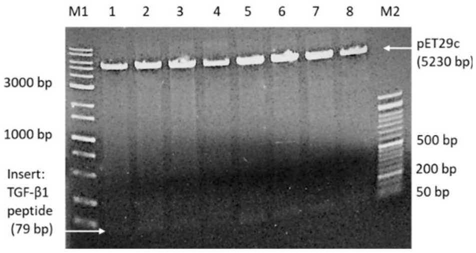

(**a**)

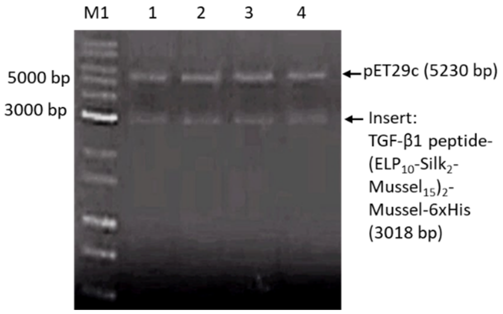

(**b**)

Figure S1. Synthesis of the gene "TGF-β1 peptide-(ELP_{10}-$Silk_2$-$Mussel_{15}$)$_2$-Mussel-6xHis". a) Electrophoresis in 1% w/v agarose gel of pET29c and "TGF-β1 peptide" insert after digestion of the recombinant plasmid "pET29c-TGF-β1 peptide" with NdeI and XhoI. Lanes 1-8: plasmids from different colonies that were screened. After DNA sequencing, it was verified that all colonies contained plasmids with the desired insert. b) Assembly of the gene "TGF-β1 peptide-(ELP_{10}-$Silk_2$-$Mussel_{15}$)$_2$-Mussel-6xHis" by PRe-RDL. Lanes 1-4: plasmids from different colonies that were screened by digestion with NdeI and XhoI. M1: 1 kb DNA ladder, M2: 50 bp DNA ladder.

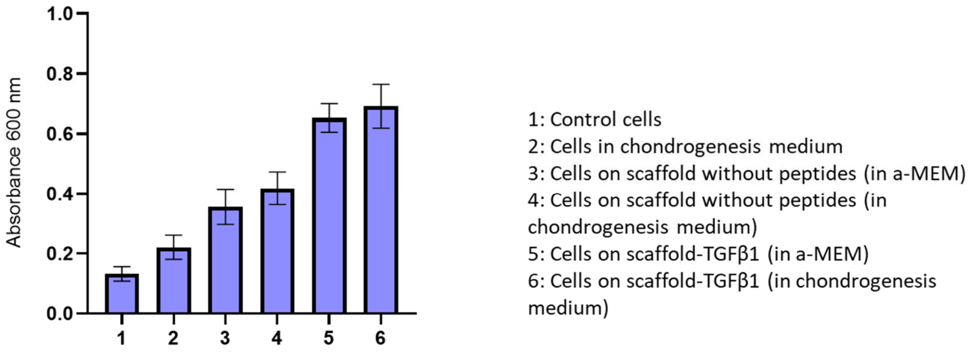

Figure S2. Quantification of Alcian Blue staining at 21 days. Optical absorption was measured at 600 nm with a microplate reader (Biotek Plate Reader).

Tables

Table S1. Nucleotide and amino acid sequences of the building blocks.

Building block	Nucleotide sequence (5′ to 3′)	Amino acid sequence
ELP	GTGCCGGGCGTTGGTGTTCCGGGCGTCGGTGTCCCGGGTAAAGGCGTTCCGGGTGTTGGTGTTCCGGGTGTTGGC	VPGVGVPGVGVPGKGVPGVGVPGVG
Silk	GTGGGCGCGGGTGCCGGCAGCGGTGCAGGCGCTGGTTCTGGCGCTGGCGCGGGTTCCGGTGCCGGTGCAGGCTCCGGCGCCGGCGCTGGTTCTGGC	V(GAGAGS)$_5$G
Mussel	GCGAAACCGAGCTATCCGCCGACCTATAAA	AKPSYPPTYK
Fibronectin (RGD) peptide	TATGCGGTGACCGGTCGTGGTGATAGCCCGGCCTCTAGCGGC	YAVTGRGDSPASSG
Laminin peptide	TATCATTACGTGACCATTACGCTGGATTTACAGCAA	YHYVTITLDLQQ
Heparin-binding peptide	TATCCGACCCAGCGTGCGCGCTATCAATGGGTGCGTTGCAACCCG	YPTQRARYQWVRCNP
TGF-β1 peptide	TATTACGTGGGCCGTAAACCGAAA	YYVGRKPK
6xHis tag	CATCACCATCACCATCAC	HHHHHH

Table S2. Primer sequences for the synthesis of the DNA sequence encoding the “TGF-β1 peptide” by PCR.

Primer name	Sequence (5′-3′)
TGF-β1 peptide forward	GACTGTCATATGGAAAGCCTGTTGCCGGTGAGGAGCGGTGTGGGCTATTACGTGGGCCGTAAACCGAAA
TGF-β1 peptide reverse	AACCACCTCGAGCTGAAGAGCTGTGGTCTGGTGCCTTTCGGTTTACGGCCCACGTAATA

Table S3. Composition of PCR reaction for the synthesis of the DNA sequence encoding the "TGF-β1 peptide".

Reagent	Final concentration
Forward primer	500 nM
Reverse primer	500 nM
Thermopol Buffer	1X (contains 2mM $MgSO_4$)
$MgSO_4$	10 mM
dNTP mix	3 mM each
Deep Vent DNA polymerase	6 U
Sterile ddH_2O	Up to 50 μL

Table S4. Conditions of PCR reaction for the synthesis of the DNA sequence encoding the "TGF-β1 peptide".

<table>
<tr><th>Stage</th><th>Temperature (°C)</th><th>Duration (min)</th><th>Number of cycles</th></tr>
<tr><td>Initial denaturation</td><td>95</td><td>3</td><td>1</td></tr>
<tr><td>Denaturation</td><td>95</td><td>1</td><td rowspan="4">20</td></tr>
<tr><td rowspan="2">Annealing</td><td>65</td><td>1</td></tr>
<tr><td>59</td><td>1</td></tr>
<tr><td>Extension</td><td>72</td><td>1</td></tr>
<tr><td>Final extension</td><td>72</td><td>5</td><td>1</td></tr>
<tr><td>Hold</td><td>4</td><td>∞</td><td></td></tr>
</table>

Table S5. Primer sequences for real-time PCR.

Primer	Nucleotide sequence (5′to3′)
GAPDH forward	GCACCGTCAAGGCTGAGAAC
GAPDH reverse	TGGTGAAGACGCCAGTGGA
RPLPO forward	AATGTGGGCTCCAAGCAGAT
RPLPO reverse	TGAGGCAGCAGTTTCTCCAG
SOX9 forward	GGCAAGCTCTGGAGACTTCTG
SOX9 reverse	CCCGTTCTTCACCGACTTCC
COL2A1 forward	AGAATCCATCTGAGAATATGC
COL2A1 reverse	CCTCTTACTGCTATACCTTTAC

ACAN forward	GGAAGGGAGGGGAACCATTG
ACAN reverse	TGATGGCTGTCCACTGACAC
TGFBR1A forward	AGCAGCAGACAATAAAGACAATGG
TGFBR1A reverse	TGTGAAGATGGGCAAGACCG
TGFBR2 forward	CTGTAATGCAGTGGGAGAAGTAAAA
TGFBR2 reverse	AATTTCTGGTCGCCCTCGAT
COL1A1 forward	CAGTGTGGCCCAGAAGAACT
COL1A1 reverse	CCGCCATACTCGAACTGGAAT
Osteocalcin forward	CGGTGCAGAGTCCAGCAAA
Osteocalcin reverse	GGTAGCGCCTGGGTCTCTTC
MMP9 forward	TTCCAGTACCGAGAGAAAGCCTAT
MMP9 reverse	GGTCACGTAGCCCACTTGGT
MMP13 forward	AAGGAGCATGGCGACTTCT
MMP13 reverse	TGGCCCAGGAGGAAAAGC
COL10A1 forward	AGAATCCATCTGAGAATATGC
COL10A1 reverse	CCTCTTACTGCTATACCTTTAC
PCNA forward	TTAAATTGTCACAGACAAGTAATGTCG
PCNA reverse	TGGCTTTTGTAAAGAAGTTCAGGTAC
BAX forward	GGGCCCACCAGCTCTGA
BAX reverse	CCTGCTCGATCCTGGATGA
BCL2 forward	CTTGACAGAGGATCATGCTGTAC
BCL2 reverse	GGATGCTTTATTTCATGAGGC
IL1b forward	ATCACTGAACTGCACGCTCC
IL1b reverse	TTGTTCTCCATATCCTGTCCC
TNFa forward	CCTCTCTCAATCAGCCCTCTG
TNFa reverse	GAGGACCTGGGAGTAGATGAG

Sequences

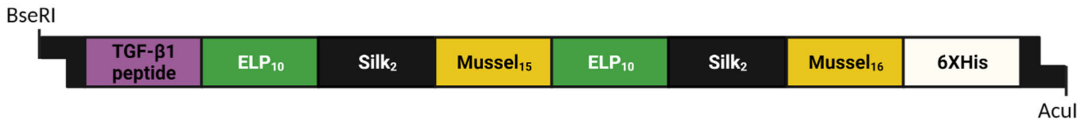

Nucleotide sequence (2952 bp):

ATGGTTAGCCTGTTGCCGGTGAGGAGCGGTGTGGGCTATTACGTGGGCCGTA
AACCGAAAGTGCCGGGCGTTGGTGTTCCGGGCGTCGGTGTCCCGGGTAAAGG
CGTTCCGGGTGTTGGTGTTCCGGGTGTTGGCGTGCCGGGCGTTGGTGTTCCGG
GCGTCGGTGTCCCGGGTAAAGGCGTTCCGGGTGTTGGTGTTCCGGGTGTTGGC
GTGCCGGGCGTTGGTGTTCCGGGCGTCGGTGTCCCGGGTAAAGGCGTTCCGG
GTGTTGGTGTTCCGGGTGTTGGCGTGCCGGGCGTTGGTGTTCCGGGCGTCGGT
GTCCCGGGTAAAGGCGTTCCGGGTGTTGGTGTTCCGGGTGTTGGCGTGCCGG
GCGTTGGTGTTCCGGGCGTCGGTGTCCCGGGTAAAGGCGTTCCGGGTGTTGGT
GTTCCGGGTGTTGGCGTGCCGGGCGTTGGTGTTCCGGGCGTCGGTGTCCCGGG
TAAAGGCGTTCCGGGTGTTGGTGTTCCGGGTGTTGGCGTGCCGGGCGTTGGTG
TTCCGGGCGTCGGTGTCCCGGGTAAAGGCGTTCCGGGTGTTGGTGTTCCGGGT
GTTGGCGTGCCGGGCGTTGGTGTTCCGGGCGTCGGTGTCCCGGGTAAAGGCG
TTCCGGGTGTTGGTGTTCCGGGTGTTGGCGTGCCGGGCGTTGGTGTTCCGGGC
GTCGGTGTCCCGGGTAAAGGCGTTCCGGGTGTTGGTGTTCCGGGTGTTGGCGT
GCCGGGCGTTGGTGTTCCGGGCGTCGGTGTCCCGGGTAAAGGCGTTCCGGGT
GTTGGTGTTCCGGGTGTTGGCGTGGGCGCGGGTGCCGGCAGCGGTGCAGGCG
CTGGTTCTGGCGCTGGCGCGGGTTCCGGTGCCGGTGCAGGCTCCGGCGCCGG
CGCTGGTTCTGGCGTGGGCGCGGGTGCCGGCAGCGGTGCAGGCGCTGGTTCT
GGCGCTGGCGCGGGTTCCGGTGCCGGTGCAGGCTCCGGCGCCGGCGCTGGTT
CTGGCGCGAAACCGAGCTATCCGCCGACCTATAAAGCCAAACCGTCTTATCC
GCCGACGTATAAAGCAAAACCGAGCTATCCGCCGACGTATAAAGCGAAACC

GAGCTATCCGCCGACCTATAAAGCCAAACCGTCTTATCCGCCGACGTATAAA
GCAAAACCGAGCTATCCGCCGACGTATAAAGCGAAACCGAGCTATCCGCCG
ACCTATAAAGCCAAACCGTCTTATCCGCCGACGTATAAAGCAAAACCGAGCT
ATCCGCCGACGTATAAAGCGAAACCGAGCTATCCGCCGACCTATAAAGCCA
AACCGTCTTATCCGCCGACGTATAAAGCAAAACCGAGCTATCCGCCGACGTA
TAAAGCGAAACCGAGCTATCCGCCGACCTATAAAGCCAAACCGTCTTATCCG
CCGACGTATAAAGCAAAACCGAGCTATCCGCCGACGTATAAAGTGCCGGGC
GTTGGTGTTCCGGGCGTCGGTGTCCCGGGTAAAGGCGTTCCGGGTGTTGGTGT
TCCGGGTGTTGGCGTGCCGGGCGTTGGTGTTCCGGGCGTCGGTGTCCCGGGTA
AAGGCGTTCCGGGTGTTGGTGTTCCGGGTGTTGGCGTGCCGGGCGTTGGTGTT
CCGGGCGTCGGTGTCCCGGGTAAAGGCGTTCCGGGTGTTGGTGTTCCGGGTGT
TGGCGTGCCGGGCGTTGGTGTTCCGGGCGTCGGTGTCCCGGGTAAAGGCGTT
CCGGGTGTTGGTGTTCCGGGTGTTGGCGTGCCGGGCGTTGGTGTTCCGGGCGT
CGGTGTCCCGGGTAAAGGCGTTCCGGGTGTTGGTGTTCCGGGTGTTGGCGTGC
CGGGCGTTGGTGTTCCGGGCGTCGGTGTCCCGGGTAAAGGCGTTCCGGGTGTT
GGTGTTCCGGGTGTTGGCGTGCCGGGCGTTGGTGTTCCGGGCGTCGGTGTCCC
GGGTAAAGGCGTTCCGGGTGTTGGTGTTCCGGGTGTTGGCGTGCCGGGCGTTG
GTGTTCCGGGCGTCGGTGTCCCGGGTAAAGGCGTTCCGGGTGTTGGTGTTCCG
GGTGTTGGCGTGCCGGGCGTTGGTGTTCCGGGCGTCGGTGTCCCGGGTAAAG
GCGTTCCGGGTGTTGGTGTTCCGGGTGTTGGCGTGCCGGGCGTTGGTGTTCCG
GGCGTCGGTGTCCCGGGTAAAGGCGTTCCGGGTGTTGGTGTTCCGGGTGTTGG
CGTGGGCGCGGGTGCCGGCAGCGGTGCAGGCGCTGGTTCTGGCGCTGGCGCG
GGTTCCGGTGCCGGTGCAGGCTCCGGCGCCGGCGCTGGTTCTGGCGTGGGCG
CGGGTGCCGGCAGCGGTGCAGGCGCTGGTTCTGGCGCTGGCGCGGGTTCCGG
TGCCGGTGCAGGCTCCGGCGCCGGCGCTGGTTCTGGCGCGAAACCGAGCTAT
CCGCCGACCTATAAAGCCAAACCGTCTTATCCGCCGACGTATAAAGCAAAA
CCGAGCTATCCGCCGACGTATAAAGCGAAACCGAGCTATCCGCCGACCTATA
AAGCCAAACCGTCTTATCCGCCGACGTATAAAGCAAAACCGAGCTATCCGC
CGACGTATAAAGCGAAACCGAGCTATCCGCCGACCTATAAAGCCAAACCGT
CTTATCCGCCGACGTATAAAGCAAAACCGAGCTATCCGCCGACGTATAAAGC
GAAACCGAGCTATCCGCCGACCTATAAAGCCAAACCGTCTTATCCGCCGACG

TATAAAGCAAAACCGAGCTATCCGCCGACGTATAAAGCGAAACCGAGCTAT
CCGCCGACCTATAAAGCCAAACCGTCTTATCCGCCGACGTATAAAGCAAAA
CCGAGCTATCCGCCGACGTATAAAGCGAAACCGAGCTATCCGCCGACCTATA
AAGCGAAACCGAGCTATCCGCCGACCTATAAAGGCAGCGGTTCTGGTGGCTT
CAGCCATCACCATCACCATCACTAATAA

Amino acid sequence (982 aa):

MVSLLPVRSGVGYYVGRKPKVPGVGVPGVGVPGKGVPGVGVPGVGVPGVGVPG
VGVPGKGVPGVGVPGVGVPGVGVPGVGVPGKGVPGVGVPGVGVPGVGVPGVG
VPGKGVPGVGVPGVGVPGVGVPGVGVPGKGVPGVGVPGVGVPGVGVPGVGVP
GKGVPGVGVPGVGVPGVGVPGVGVPGKGVPGVGVPGVGVPGVGVPGVGVPGK
GVPGVGVPGVGVPGVGVPGVGVPGKGVPGVGVPGVGVPGVGVPGVGVPGKGV
PGVGVPGVGVGAGAGSGAGAGSGAGAGSGAGAGSGAGAGSGVGAGAGSGAG
AGSGAGAGSGAGAGSGAGAGSGAKPSYPPTYKAKPSYPPTYKAKPSYPPTYKAKP
SYPPTYKAKPSYPPTYKAKPSYPPTYKAKPSYPPTYKAKPSYPPTYKAKPSYPPTYKA
KPSYPPTYKAKPSYPPTYKAKPSYPPTYKAKPSYPPTYKAKPSYPPTYKAKPSYPPTY
KVPGVGVPGVGVPGKGVPGVGVPGVGVPGVGVPGVGVPGKGVPGVGVPGVGV
PGVGVPGVGVPGKGVPGVGVPGVGVPGVGVPGVGVPGKGVPGVGVPGVGVPG
VGVPGVGVPGKGVPGVGVPGVGVPGVGVPGVGVPGKGVPGVGVPGVGVPGVG
VPGVGVPGKGVPGVGVPGVGVPGVGVPGVGVPGKGVPGVGVPGVGVPGVGVP
GVGVPGKGVPGVGVPGVGVPGVGVPGVGVPGKGVPGVGVPGVGVGAGAGSGA
GAGSGAGAGSGAGAGSGAGAGSGVGAGAGSGAGAGSGAGAGSGAGAGSGAG
AGSGAKPSYPPTYKAKPSYPPTYKAKPSYPPTYKAKPSYPPTYKAKPSYPPTYKAKP
SYPPTYKAKPSYPPTYKAKPSYPPTYKAKPSYPPTYKAKPSYPPTYKAKPSYPPTYKA
KPSYPPTYKAKPSYPPTYKAKPSYPPTYKAKPSYPPTYKAKPSYPPTYKAKPSYPPTY
KGSGSGGFSHHHHHH
